# A contemporary baseline record of the world’s coral reefs

**DOI:** 10.1038/s41597-020-00698-6

**Published:** 2020-10-20

**Authors:** Alberto Rodriguez-Ramirez, Manuel González-Rivero, Oscar Beijbom, Christophe Bailhache, Pim Bongaerts, Kristen T. Brown, Dominic E. P. Bryant, Peter Dalton, Sophie Dove, Anjani Ganase, Emma V. Kennedy, Catherine J. S. Kim, Sebastian Lopez-Marcano, Benjamin P. Neal, Veronica Z. Radice, Julie Vercelloni, Hawthorne L. Beyer, Ove Hoegh-Guldberg

**Affiliations:** 1 Global Change Institute, The University of Queensland, St Lucia, QLD 4072 Australia; 2 School of Biological Sciences, The University of Queensland, St Lucia, QLD 4072 Australia; 3 Australian Institute of Marine Science, Cape Cleveland, QLD 4810 Australia; 4 Australian Research Council (ARC) Centre for Excellence for Coral Reef Studies at The University of Queensland, St Lucia, QLD 4072 Australia; 5 Berkeley Artificial Intelligence Research, University of California, Berkeley, CA 94720 USA; 6 Underwater Earth, Sydney, NSW Australia; 7 California Academy of Sciences, San Francisco, CA 94118 USA; 8 Remote Sensing Research Centre, School of Earth and Environmental Sciences, The University of Queensland, St Lucia, QLD 4072 Australia; 9 Australian Rivers Institute, Griffith University, Gold Coast, QLD 4222 Australia; 10 Colby College, Environmental Studies Department, Waterville, Maine, ME 04901 USA; 11 Bigelow Laboratory for Ocean Sciences, East Boothbay, ME 04544 USA; 12 ARC Centre of Mathematical and Statistical Frontiers, Queensland University of Technology, Brisbane, QLD 4000 Australia; 13 School of Mathematical Sciences, Science and Engineering Faculty, Queensland University of Technology, Brisbane, QLD 4000 Australia

**Keywords:** Ecosystem ecology, Conservation biology, Marine biology

## Abstract

Addressing the global decline of coral reefs requires effective actions from managers, policymakers and society as a whole. Coral reef scientists are therefore challenged with the task of providing prompt and relevant inputs for science-based decision-making. Here, we provide a baseline dataset, covering 1300 km of tropical coral reef habitats globally, and comprised of over one million geo-referenced, high-resolution photo-quadrats analysed using artificial intelligence to automatically estimate the proportional cover of benthic components. The dataset contains information on five major reef regions, and spans 2012–2018, including surveys before and after the 2016 global bleaching event. The taxonomic resolution attained by image analysis, as well as the spatially explicit nature of the images, allow for multi-scale spatial analyses, temporal assessments (decline and recovery), and serve for supporting image recognition developments. This standardised dataset across broad geographies offers a significant contribution towards a sound baseline for advancing our understanding of coral reef ecology and thereby taking collective and informed actions to mitigate catastrophic losses in coral reefs worldwide.

## Abstract (summary)

**Table:** 

Measurement(s)	ecosystem • coral reef • composition
Technology Type(s)	automated image annotation • machine learning
Factor Type(s)	year of data collection • geographic location
Sample Characteristic - Organism	Anthozoa • Algae • Porifera
Sample Characteristic - Environment	marine coral reef biome • marine coral reef fore reef
Sample Characteristic - Location	Atlantic Ocean • Eastern Australia • Indian Ocean • Southeast Asia • Pacific Ocean • Great Barrier Reef

Machine-accessible metadata file describing the reported data: 10.6084/m9.figshare.13007516

## Background & Summary

The escalating deterioration of coral reefs over the past half-century^1,2^ has imposed urgent challenges for coral reef science^3,4^, especially at the spatial and temporal scales^5^ required to support science-based management and conservation at global scales^6,7^. Novel research and management approaches that consider current ecological and socio-cultural paradigms of coral reefs are therefore pivotal for addressing such challenges^4,7–13^. Many of these strategies, however, face major challenges in terms of data acquisition, analysis and implementation^14^. In addition, the reluctance to share data among scientists^15^ may often frustrate progress in reef science, management and conservation^16^. Clearly this increasing demand of scientific outputs needs to be pursued collectively as a top priority^16^ in order to take up the challenges of managing Anthropocene reefs effectively in a rapidly changing world^4–6,17^.

There have been a large number of technological innovations that are revolutionising the collection and analysis of critical data^18–23^. Yet, the lack of adequate metrics for monitoring^3^, the paucity of large-scale assemblage datasets^7^ and, limited availability of key long-term ecological data^24^ have generated a lag in the task of incorporating scientific findings into management and policy actions. Large-scale and standardised field data have been recognised as essential information for tackling the scale of problems and potential solutions^3,6,7,25,26^. Worldwide data collection and analysis have constrained our ability to detect changes at global scales^27,28^, partly due to our limited understanding of future reef responses and management options at global-scales. Regional and sub-regional analyses have provided the most significant insights of contemporary coral reef dynamics and the implicated drivers. These studies, however, had to compile, filter and aggregate multiple small-scale data sets based on different methods (e.g. transects lines, chain, photo-quadrats)^29–33^. These meta-analyses are indispensable, but the time-intensive approach can be minimised in the future.

Here, we present a standardised and contemporary archive of baseline images and data^34^ of the world’s tropical coral reefs between 2012 to 2018. The dataset overcomes limitations and satisfies requirements of some aforementioned data needs in terms of scale, extent, and standardisation for advancing reef science and management. The imagery and ecological data were produced within the framework of the XL Catlin Seaview Survey Project^35^. The dataset includes over one million standardised and geo-referenced photo-quadrat images, each one covering approximately 1 m^2^ of substrate. These images were taken at 860 reef locations within the Western Atlantic (Caribbean, Lucayan Archipelago, and Bermuda), the Central Indian Ocean (Maldives and Chagos Archipelagos), the Central Pacific Ocean (Hawaii), the Coral Triangle and Southeast Asia (Indonesia, Philippines, Timor-Leste, Solomon Islands, and Taiwan), and Eastern Australia (Great Barrier Reef). The locations were surveyed using an underwater propulsion vehicle customised with high-definition cameras along 1.5 to 2.0 km transects at a standard depth of 10 m (±2 m), covering unparalleled areas of coral reefs (over 1300 km of fore-reef slopes). Multi-temporal images exist for some reef locations before and after the 2016 global mass bleaching event in the Great Barrier Reef, Indonesia, Maldives, Philippines, Hawaii and Taiwan.

The dataset provides estimates of the areal extent of main benthic groups (e.g., “hard coral”, “algae”, etc.) at each reef location and for more specific categories for each photo-quadrat. These estimations were obtained from the application of an automated image annotation method (Deep Learning Convolutional Neural Networks)^23^. Validation of this approach showed an unbiased 97% agreement between human and automated annotations, with errors of the automated estimations ranging between <2% and 7%^23^.

The dataset offers exceptional opportunities for analysis across multiple spatial scales from local to global, enabling the potential detection of change while providing a baseline by which to investigate the contemporary decline and recovery of coral reefs, and addressing a range of broad and specific ecological questions. Furthermore, the dataset offers human-classified annotations and images that can be used to train and validate automated image classifiers. The dataset and imagery have contributed to a substantial number of publications^23,25,35–39^. The ultimate goal of this record is to expedite scientific investigation and evidence-based management. Given the remoteness of some surveyed locations, broad geographic representation as well as detailed spatial and taxonomic resolution, the data provide a unique resource to further advance our understanding of ecological responses across a range of changing pressures and build a stronger base knowledge that supports conservation actions.

## Methods

A comprehensive description of the methodological aspects used during the field surveys and image analysis have been published in González-Rivero *et al*.^23,25,35^. Therefore, here we include a synopsis of how this dataset was generated and made available to the wider community.

**Fig. 1:**
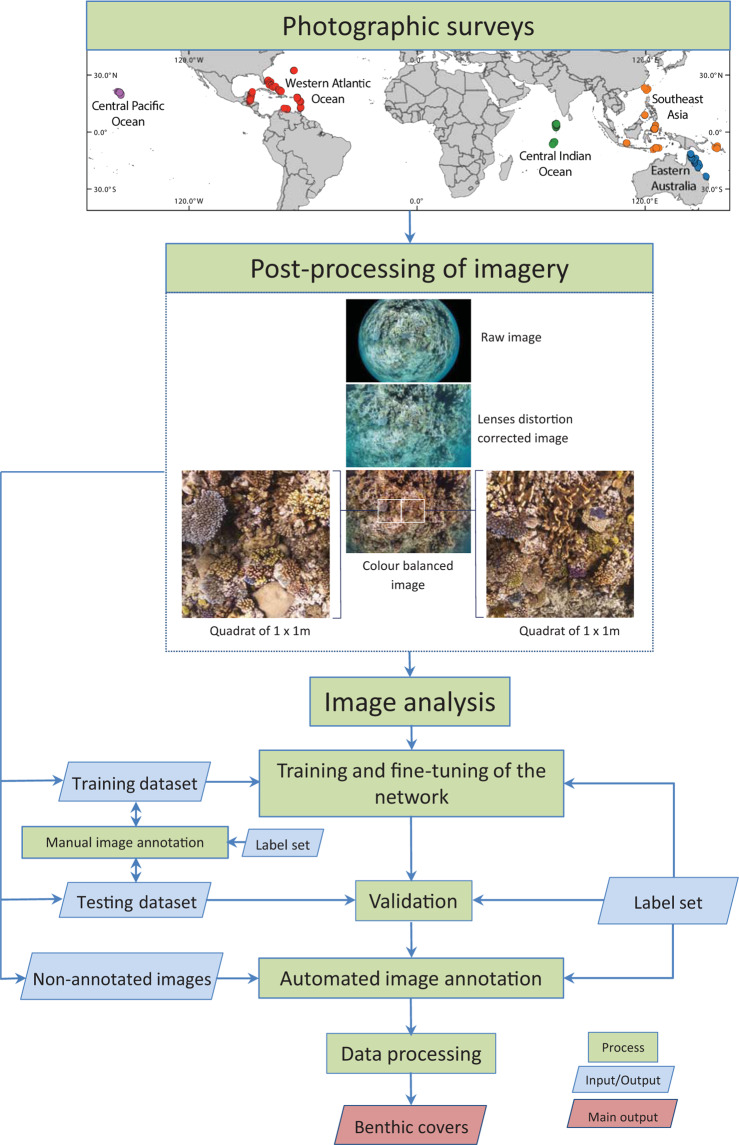
The workflow for generating the global dataset of coral reef imagery and associated data. The 860 photographic surveys from the Western Atlantic Ocean, Southeast Asia, Central Pacific Ocean, Central Indian Ocean, and Eastern Australia, were conducted between 2012 and 2018. Reef locations are represented by points colour-coded according to the survey region. Surveys images were post-processed in order to transform raw fish-eye images into 1 × 1 m quadrats for manual and automated annotation (inset originally published in González-Rivero *et al*.^23^ as Figure S1). For the image analysis, nine networks were trained. For each network, images were divided in two groups: Training and Testing images. Both sets were manually annotated to create a training dataset and verification dataset. The training dataset was used to train and fine-tune the network. The fully trained network was then used to classify the test images, and contrast the outcomes (Machine) against the human annotations (Observer) in the test dataset during the validation process. Finally, the non-annotated images (photo-quadrats) were automatically annotated using the validated network. The automated classifications were processed to originate the benthic covers that constitute this dataset. QGIS software was used to generate the map using the layer “Countries WGS84” downloaded from ArcGIS Hub (http://hub.arcgis.com/datasets/UIA::countries-wgs84).

Our approach involved the rapid acquisition of high-resolution imagery over large extent of reefs and efficient image analysis to provide key information about the state of coral reef benthic habitat across multiple spatial scales^23^. The data generation and processing involved three main components: (1) photographic surveys, (2) post-processing of images and (3) image analysis, which are described and summarised below in Fig. 1.

### Photographic surveys

An underwater propulsion vehicle customised with a camera system (“SVII”, Supplementary Fig. 1), consisting of three synchronised DSLR (Digital Single-Lens Reflex) cameras (Cannon 5D-MkII cameras and Nikon Fisheye Nikkor lens with 10.5 mm focal length), was used to survey the fore-reef (reef slope) habitats from five major coral reef regions: Central Pacific Ocean, Western Atlantic Ocean, Central Indian Ocean, Southeast Asia and Eastern Australia in 23 countries or territories (Table 1, Supplementary Fig. 2). Within each region, multiple reef locations were surveyed aiming to capture the variability and status of fore-reefs environments across regions and within each region. Sampling design varied according to particular environmental and socioeconomic factors potentially influencing the distribution and structure of coral reef assemblages at each region and/or country. Overall, prior to field expeditions, reef localities were selected considering factors such as wave exposure, reef zones (i.e. fore-reefs), local anthropogenic stressors (e.g. coastal development), fishing pressures, levels of management (e.g. marine park, protected areas), and presence of monitoring sites.

**Table 1:** Summary of the photographic surveys conducted between 2012 and 2018.

Region	Survey year	Country or Territory	Surveys	Quadrats
Western Atlantic Ocean	2013	Anguilla	15	22,250
	2013	Aruba	3	5,615
	2013	The Bahamas	30	42,678
	2013	Belize	24	32,515
	2013	Bermuda	12	25,748
	2013	Bonaire	13	23,028
	2013	Curacao	16	29,419
	2013	Guadeloupe	14	15,277
	2013	Mexico	23	38,192
	2013	Saint Martin	4	4,368
	2013	Saint Vincent and the Grenadines	25	25,498
	2013	Saint Eustatius	3	2,033
	2013	Turks and Caicos Islands	13	18,387
Eastern Australia	2012, 2014,2016,2017	Australia (Great Barrier Reef)	261	316,369
Central Indian Ocean	2015	The Chagos Archipelago	29	42,777
	2015, 2017	Maldives	63	94,921
Southeast Asia	2016, 2018	Taiwan	29	30,062
	2014	Timor-Leste	26	28,482
	2014, 2018	Indonesia	114	124,889
	2014, 2018	The Philippines	24	33,038
	2014	The Solomon Islands	20	33,667
Central Pacific Ocean	2015, 2016	United States	99	93,111
Totals		**23**	**860**	**1,082,324**

Underwater images were collected in each reef location once every three seconds, approximately every 2 m apart, following a transect along the seascape at a standard depth of 10 m (±2 m). Although overlap between consecutive images is possible, the process for extracting standardised photo-quadrats from an image ensures that the photo-quadrats are non-overlapping between and within images (see further details next section). Each transect averaged 1.8 km in length, hereafter referred to as a “survey”. See Supplementary Fig. 3 for an explanation of the hierarchical structure of the photographic surveys. No artificial illumination was used during image capture, but light exposure was manually adjusted by modifying the ISO during the dive, using an on-board tablet computer encased in an underwater housing (Supplementary Fig. 1). This computer enabled the diver to control camera settings (exposure and shutter speed) according to light conditions. Images were geo-referenced using a surface GPS unit tethered to the diver (Supplementary Fig. 1). Altitude and depth of the camera relative to the reef substrate and surface were logged at half-second intervals using a Micron Tritech transponder (altitude, Supplementary Fig. 1) and pressure sensor (depth) in order to select the imagery within a particular depth and to scale and crop the images during the post-processing stage. Further details about the photographic surveys are provided in González-Rivero *et al*.^25,35^.

### Post-processing of images for manual and automated annotation

The post-processing pipeline produced images with features required for manual and automated annotation in terms of size and appearance. The process involved several steps that transformed the raw images from the downward facing camera into photo-quadrats of 1 m^2^, hereafter referred to as a “quadrat” (Fig. 1). As imagery was collected without artificial light using a fisheye lens, each image was processed prior to annotation in order to balance colour and to correct the non-linear distortion introduced by the fisheye lens^23^ (Fig. 1). Initially, colour balance and lens distortion correction were manually applied on the raw images using Photoshop (Adobe Systems, California, USA). Later, in order to optimise the manual post-processing time of thousands of images, an automatic batch processing was conducted on compressed images^23^ (jpeg format) using Photoshop and ImageMagick, the latter an open-source software for image processing (https://imagemagick.org/index.php). In addition, using the geometry of the lens and altitude values, images were cropped to a standardised area of approximately 1 m^2^ of substrate^23,35^ (Fig. 1). Thus, the number of nonoverlapping quadrats extracted from one single raw image varied depending on the distance between the camera and the reef surface. Figure 1 illustrates a situation where the altitude of the camera allowed for the extraction of two quadrats from one raw image. Further details about colour balance and lens distortion correction and cropping are provided in González-Rivero *et al*.^23,35^.

### Image analysis: manual and automated annotation for estimating covers of benthic categories

Manual annotation of the benthic components by a human expert took at least 10 minutes per quadrat, creating a bottleneck between image post-processing and the required data-product. To address this issue, we developed an automated image analysis to identify and estimate the relative abundance of benthic components such as particular types of corals, algae, and other organisms as well as non-living components. To do this, automated image annotation based on deep learning methods (Deep Learning Convolutional Neural Networks)^23^ were applied to automatically identify benthic categories from images based on training using human annotators (manual annotation). The process for implementing a Convolutional Neural Network (hereafter “network”) and classify coral reef images implied three main stages: (i) label-set (benthic categories) definition, (ii) training and fine-tuning of the network, and (iii) automated image annotation and data processing.

#### Label-set definition

As a part of the manual and automated annotation processes to extract benthic cover estimates, label-sets of benthic categories were established based on their functional relevance to coral reef ecosystems and their features to be reliably identified from images by human annotators^25^. The labels were derived, modified and/or simplified from existing classification schemes^40,41^, and were grouped according to the main benthic groups of coral reefs including hard coral, soft coral, other invertebrates, algae, and other. Since coral reef assemblages vary in species composition at global and regional scales, and surveys were conducted at different times between 2012 and 2018 across the regions, nine label-sets accounted for such biogeographical and temporal disparity. In general, a label-set was developed after each main survey expedition to a specific region. The label-sets varied in complexity (from 23 to 61 labels), considering the differential capacity to visually recognise (in photographs) corals to the lowest possible taxon between the regions. While label-sets for the Atlantic and Central Pacific (Hawaii) included categories with coral genus and species, for the Indian Ocean (Maldives, Chagos Archipelago), Southeast Asia (Indonesia, Philippines, Timor-Leste, Solomon Islands, and Taiwan), and Eastern Australia, corals comprised labels based on a combination of taxonomy (e.g., family and genus) and colony morphology (e.g., branching, massive, encrusting, foliose, tabular).

The other main benthic groups were generally characterised by labels reflecting morphology and/or functional groups across the regions. “Soft Corals” were classified into three groups: 1) Alcyoniidae (soft corals), the dominant genera; 2) Sea fans and plumes from the family Gorgoniidae; and 3) Other soft corals. “Algae” groups were categorised according to their functional relevance: 1) Crustose coralline algae; 2) Macroalgae; and 3) Epilithic Algal Matrix. The latter is a multi-specific algal assemblage smothering the reef surface of up to 1 cm in height (dominated by algal turfs). “Other Invertebrates” consisted of labels to classify sessile invertebrates different to soft corals (e.g., *Millepora*, bryozoans, clams, tunicates, soft hexacorrallia, hydroids) and some mobile invertebrates observed in the images (mostly echinoderms). The remaining group, “Other”, consisted of sand, sediments, and occasional organisms or objects detected in the images such as fish, human debris (e.g., plastic, rope, etc.), and transect hardware. The exception within these main groups were the “Sponges”, which were classified and represented by multiple labels only in the Atlantic (given their abundance and diversity in the Caribbean), including categories with sponge genus and species, and major growth forms (rope, tube, encrusting, massive).

#### Training and fine-tuning of the network

The deep learning approach used relies on a convolutional neural network architecture named VGG-D 16^42^. Details on the initialisation and utilisation of this network are provided in González-Rivero *et al*.^23^. A total of nine networks were used, one for each country within the regions, except for the Western Atlantic Ocean, where the network was trained using data from several countries, and the Philippines and Indonesia, where the network was trained using data from those two countries. (Table 2). The first step in implementing a network was to randomly select a subset of images from the whole regional set to be classified, which were then divided into training and testing sets (Fig. 1). Human experts manually annotated both sets using the corresponding label-set under CoralNet^43^, an online platform designed for image analysis of coral reef related materials (https://coralnet.ucsd.edu/). The number of images and points manually annotated per network is presented in Table 2 (generally 100 points per image for training sets and 40 or 50 points per image for testing sets).

**Table 2:** Summary of the images, manual point annotations, and test transects used during the train and test processes of each network.

Trained network	Region	Folder name at the Repository	Country or Territory	Training images	Training annotations	Testing images	Testing annotations	Test transects
1	WAO	ATL	Anguilla	10	44,900	50	48,000	5
			Aruba	7		30		3
			The Bahamas	70		150		12
			Belize	111		115		13
			Bermuda	17		60		8
			Bonaire	21		115		8
			Curacao	36		90		7
			Guadeloupe	17		75		6
			Mexico	60		115		11
			Saint Martin	9		25		2
			Saint Vincent and the Grenadines	54		60		7
			Saint Eustatius	12		25		1
			Turks and Caicos Islands	25		50		4
2	CIO	IND_CHA	Chagos	359	35,900	331	16,550	29
3	CIO	IND_MDV	Maldives	1,171	117,100	540	27,000	52
4	EA	PAC_AUS	Australia	1,234	129,340	1,426	57,080	130
5	CPO	PAC_USA	Unitated States (Hawaii)	501	50,100	660	33,000	60
6	SEA	PAC_IDN_PHL	Indonesia	614	75,100	600	45,000	50
			Philippines	137		300		24
7	SEA	PAC_SLB	The Solomon Islands	439	44,200	300	15,000	29
8	SEA	PAC_TWN	Taiwan	350	35,000	300	15,000	27
9	SEA	PAC_TLS	Timor-Leste	547	55,100	330	16,500	29
**Totals**				**5,801**	**586,740**	**5,747**	**273,130**	**517**

Abbreviations at Region column = Western Atlantic Ocean (WAO), Central Indian Ocean (CIO), Eastern Australia (EA), Central Pacific Ocean (CPO), Southeast Asia (SEA).

Each training and testing data set were exported from CoralNet^43^ and used along with the associated quadrats to support an independent training and fine-tuning process aimed to find the network configuration that produced the best outcomes. Initially, each quadrat used from the training and testing sets was converted to a set of patches cropped out around each annotation point location. The patch area to crop around each annotation point was set to 224 × 224 pixels to align with the pre-defined image input size of the VGG-D architecture. The fine-tuning exercise ran in general for 40 K iterations to establish the best combination of model parameters or weights that minimised the cross-entropy loss while the overall accuracy increased. An independent 20% subset from the original set of quadrats was used to assess the performance of the final classification (% of accuracy). In addition, parameters of learning rate and image scale were independently optimised for each network by running an experiment using different values for such parameters in order to select the values that derived the smallest errors per label. Further details of the model parametrisation for each network are provided in González-Rivero *et al*.^23^ (see Supplementary Material).

#### Automated image annotation and data processing

Once optimised, a network was used to automatically annotate the corresponding set of non-annotated quadrats. The quadrats were processed through the network, where for each quadrat, 50 points (input patches) were classified using the associated labels. Upon completion of automated image annotation for a specific region/country, the annotation outputs containing locations of 50 pixels (i.e., their x and y coordinates) with their associated labels per quadrat (a csv file per quadrat) were incorporated and collated into a MySQL database along with information about the field surveys. In addition to the manual and automated annotations tables (raw data), we provide two levels of aggregation for the benthic data. First, the relative abundance (cover) for each of the benthic labels per quadrat, which was calculated as the ratio between the numbers of points classified for a given label by the total number of points evaluated in a quadrat. Second, the relative abundance for each of the main benthic groups (hard coral, soft coral, other invertebrates, algae, and other) per survey, which involved three calculations: 1) summarise the quadrat covers by image averaging all the quadrats from one single image per label, 2) summarise image covers by survey averaging all the images across one survey per label, and 3) merge survey data by main benthic groups summing the covers of all labels belonging to the same group across one survey.

## Data Records

The dataset presented here has been made freely available through The University of Queensland “eSpace” repository^34^, and released under a Creative Commons Attribution license (CC BY 3.0; https://creativecommons.org/licenses/by/3.0/deed.en_US). For attribution, we expect users to cite this paper when using the content. It includes three core components: 1) a series of relational tables (csv format) with the manual and automated annotations (raw data), 2) a series of relational tables (csv format) with benthic covers per survey and quadrat (processed data), dataset IDs, and label-set descriptions, and 3) the imagery associated with the dataset (jpeg format). We are providing cover data from more than one million of quadrats (over 55 million of associated data records) based on automated annotation, and over 859 K points (image features) classified by humans within selected quadrats through the CoralNet^43^ web interface that can be used to implement approaches of automated image analyses.

A unique identification number (ID) was assigned to every survey (5 digits), image (9 digits), and quadrat (11 digits) which are the basis for the database relational links among tables and files. For example, in quadrat ID 17001644602, the first five digits correspond to the survey ID (17001), the survey ID and the next four digits (6446, a number automatically assigned by the camera between 0001 and 9999) to the image ID (170016446), and the image ID along with the last two digits (02, quadrat number within an image automatically assigned during the cropping process) to the quadrat ID. The core table “seasurvey_quadrat.csv” contains all the survey, image and quadrat IDs (over 1.1 million records) and is zipped within the “tabular-data.zip” file. See Supplementary Fig. 3 for an explanation of the hierarchical structure of the dataset.

### Manual and automated annotations

The structure of the tables containing the data records from manual (testing and training datasets) and automated annotations is presented in Table 3. Each annotation record is identified by the quadrat ID and includes the coordinates of the pixel that has been annotated (x and y), and its corresponding label. Both types of annotation records are zipped within the “tabular-data.zip” file. While there is a unique file (“seaviewsurvey_annotations.csv”) for the automated annotations with more than 55.2 million data records, there are nine files with the manual annotations, which correspond to the regions/countries conforming the training sets (Table 2). They were named “annotations_” with the region/country code (Table 3) appended (e.g., annotations_PAC_AUS.csv).

**Table 3:** List of tables/files (and their structure) included in the repository.

Table/File	Data field	Descriptor
seaviewsurvey_surveys.csv	surveyid	A five unique digit survey ID representing data collection at one location (a transect location) at one point in time. This ID is unique in this table and used in the folder naming code. There may be multiple survey IDs associated with a transect ID if multi-temporal surveys were conducted at this reef location
	transectid	The five unique digit transect ID. A transect ID will appear more than once in this field if the transect has been surveyed temporally (more than one occasion)
	surveydate	The date (YYYYMMDD) on which the survey was completed
	ocean	The three letter code representing the ocean within which the survey occurred: ATL = Atlantic Ocean, IND = Indian Ocean, PAC = Pacific Ocean
	country	The three letter country code of the Exclusive Economic Zone within which the survey occurs (e.g., AUS = Australia)
	folder_name	The full name of the zipped folder associated with the survey (e.g., “ PAC_AUS_47035_201710”) where quadrats are stored within the “photo_quadrats” folder
	lat_start	The latitude of the start of the survey (decimal degrees)
	lng_start	The longitude of the start of the survey (decimal degrees)
	lat_end	The latitude of the end of the survey (decimal degrees)
	lng_end	The longitude of the end of the survey (decimal degrees)
	pr_hard_coral	The proportional cover of hard coral
	pr_algae	The proportional cover of algae
	pr_soft_coral	The proportional cover of soft coral
	pr_oth_invert	The proportional cover of other invertebrates apart from hard corals and soft corals
	pr_other	The proportional cover of other categories apart from hard corals, algae, soft corals and other invertebrates
seaviewsurvey_quadrats.csv	surveyid	The five-digit survey ID number from the “seaviewsurvey_surveys.csv” table. There is a one:many relationship between the surveys table and the quadrats table.
	imageid	The nine-digit image ID. This correspond to the five-digit survey ID and four more digits between 0001 and 9999 (number of the picture automatically assigned by the camera). Identical image ID’s will appear multiple times in this field as one image has associated multiple quadrats
	quadratid	The 11 unique digit quadrat ID. This correspond to the nine-digit image ID and two more digits usually between 01 and 09, which refer to the quadrat number within an image (automatically assigned during the cropping process). “.jpg” must be added to the quadrat ID to derive the filename of the quadrat (with the extension) within the imagery.
seaviewsurvey_reefcover_[region].csv	surveyid	The five-digit survey ID number from the seaviewsurvey_surveys.csv table. There is a one:many relationship between the surveys table and the reefcover table.
	imageid	The nine-digit image ID from the seaviewsurvey_quadrats.csv. Identical image ID’s appear multiple times in this field as one image has associated multiple quadrats
	quadratid	The 11-digit quadrat ID from the seaviewsurvey_quadrats.csv table. There is a one:many relationship between the cover table and the annotations table because each quadrat can have many annotations
	lat	The latitude the survey (decimal degrees)
	lng	The longitude of the survey (decimal degrees)
	[label]	The proportional cover of the label within the quadrat. A description of each label is presented in the “seaviewsurvey_labelsets.csv” table
seaviewsurvey_annotations.csv	quadratid	The 11-digit quadrat ID from the seaviewsurvey_quadrats.csv table. There is a one:many relationship between the quadrat table and the annotations table because each quadrat can have many annotations
	y	Y coordinate (row) of the pixel (graphics format image, origin of first pixel in the upper left corner) that has been automatically annotated.
	x	X coordinate (column) of the pixel (graphics format image, origin of first pixel in the upper left corner) that has been automatically annotated.
	label	The short code representing the category identified. A description of each label is presented in the “seaviewsurvey_labelsets.csv” table
annotations_[ocean]_[country].csv	quadratid	The 11 digit quadrat ID from the seaviewsurvey_quadrats.csv table. There is a one:many relationship between the quadrat table and the annotations table because each quadrat can have many annotations
	y	Y coordinate (row) of the pixel (graphics format image, origin of first pixel in the upper left corner) that has been manually annotated
	x	X coordinate (column) of the pixel (graphics format image, origin of first pixel in the upper left corner) that has been manually annotated
	label_name	The full name of the label identified. A description of each label is presented in the seaviewsurvey_labelsets.csv table
	label	The short code representing the category identified. A description of each label is presented in the “seaviewsurvey_labelsets.csv” table
	func_group	Main benthic group of the label identified: hard coral, soft coral, other invertebrates, algae, other
	method	Type of annotation: random = point selected randomly during the annotation; target = point aimed during the annotation
	data_set	Application of the manual annotations: train = to train a net; test = to validate a net
seaviewsurvey_labelsets.csv	region	Name of one of the five regions surveyed: Atlantic, Indian Ocean, Pacific Australia, Southeast Asia, Pacific Hawaii
	label	The short code for one of the labels within the region considered
	func_group	Main benthic group of the label: hard coral, soft coral, other invertebrates, algae, other
	label_name	The full name of the label within the region considered
	merged_label	Alternative short code in order reduce the complexity of the label-set. Some related labels were merged for the technical validation
	merged_name	The full name of the merged label
	description_examples	A brief description of what the labels represent, with examples

The table “seaviewsurvey_labelsets.csv” provides the description of the label-sets used for each region. The structure of this table is presented in Table 3, where each record corresponds to a label within a region. Refer to the Usage Notes section for instructions on accessing visual examples of the labels.

### Benthic cover

The structure of the tables containing the data records from benthic covers per survey and quadrat is presented in Table 3. Both types of cover records are zipped within the “tabular-data.zip” file. Each data record in the surveys file (“seaviewsurvey_surveys.csv”) corresponds to a unique survey identified by the survey ID and contains the cover of the five main benthic groups (hard coral, soft coral, other invertebrates, algae, and other) along with metadata of the survey (e.g., ocean, country, latitude, longitude, etc.). Cover records per quadrat were grouped in five files and named “seaviewsurvey_reefcover_” with the ocean and/or region appended (e.g., seaviewsurvey_reefcover_pacificaustralia.csv) to facilitate the data retrieval by the main regions surveyed.

### Imagery

The imagery is grouped under three main folders within the repository^34^: (1) photo-quadrats, (2) annotated-images, and (3) survey-previews. The images files (jpeg format) associated with the quadrats cover records are stored in the “photo-quadrats” folder. To facilitate the image retrieval, quadrats are grouped by surveys in 860 zip files (.zip). Each zip folder survey was named with a code using the combination of four information components separated by underscores: the ocean in which the survey occurs (ATL = Atlantic Ocean, IND = Indian Ocean, PAC = Pacific Ocean), the three letter country code of the Exclusive Economic Zone within which the transect occurs (e.g., AUS = Australia), the unique five digit survey ID number, and the year and month of the survey formatted as YYYYMM (e.g., PAC_AUS_15007_201212.zip). Such naming convention is used in the “folder_name” data field within the “seaviewsurvey_surveys.csv” table.

The images files (jpeg format) associated with the manual annotations are stored in the “annotated-images” folder. There, images were grouped into nine zip folders according to the nine training sets of the manual annotations (Table 2). Therefore, folders were named using the corresponding annotations file name but excluding the initial part (e.g., for the “annotations_PAC_AUS.csv” file, the images folder was named as “PAC_AUS.zip”).

Low-resolution previews of all photo-quadrats per each survey (tiled into one jpeg image per survey) were zipped into archives grouped by ocean and country (e.g., “PAC_AUS” refers to the Pacific Ocean and Australia) under the “survey-previews” folder. These preview images provide a coarse overview of the content of the photo-quadrats that may help users to identify which surveys they are most interested in downloading.

## Technical Validation

The methodological approach of automated image annotation used to generate this dataset is described and validated in Gonzalez-Rivero *et al*.^23^. In order to evaluate the reliability of the estimations of benthic covers per each network we used the set of images and manual annotations defined above as testing datasets. Each testing dataset was constructed from contiguous images within standard spatial units (hereafter called “test transects”) with an extent of 30 m in length, concomitant with most coral reef monitoring programs^31,44–46^ and best represents the spatial heterogeneity within a site^25^. Thus, the aggregation of images within 30 m test transects allowed the evaluation of the performance of automated estimations within a scale that is consistent with monitoring sampling units, accounting for the variability in benthic abundance estimation among images. Test transects were selected at random while ensuring that images used for training the networks were not included. A total of 5,747 images, within 517 test transects (Table 2), were annotated (identical annotations points) by trained human observers, hereafter called “observer”, and the networks, hereafter called “machine”. The benthic composition within these test transects was averaged across images and contrasted between the two groups annotated: observer vs. machine. Specifically, we compared the error between machine and observer at the level of benthic categories and the consistency between machine and observer estimations from a community perspective.

We used the Absolute Error (E) to estimate the variability in the machine estimates when compared against observer estimations of abundance of a given benthic category. The absolute error (hereafter called “error”) for each category (*i*) was calculated as the absolute difference between the abundance estimated by the machine (*m*) and the observer (*o*; Eq. (1)). The error was calculated and compared at two aggregation levels: a) main benthic groups and b) label-set, *sensu* González-Rivero *et al*.^25^:

1 $${{\rm{E}}}_{i}=\left|{m}_{i}-{o}_{i}\right|$$

**Fig. 2:**
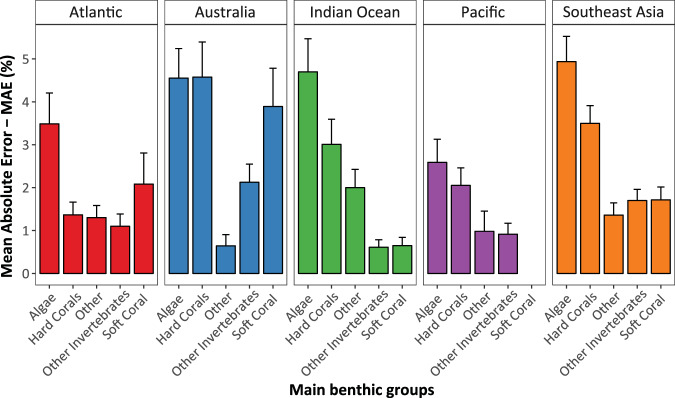
Mean Absolute Error for the automated estimation of the abundance (cover) of main benthic groups per region. Solid and error bars represent the mean and 95% Confidence Intervals of the error, respectively. Figure originally published as Fig. 3 in González-Rivero *et al*.^23^.

Such errors indicated that the trained networks (machine) were able to produce cover estimates comparable to those generated by observers, and therefore suitable for spatial and temporal analysis and monitoring^23^. While the errors are variable among regions and benthic categories (Fig. 2), they are within the range of previously reported inter- and intra-observer variability for established monitoring programs^47^ (e.g., 2–5%, Long Term Monitoring Program, AIMS, Australia). Among main benthic groups (e.g., hard coral, algae), “Algae” showed the highest errors among regions, ranging between 2% (Pacific, Hawaii) and 5% (Southeast Asia, Fig. 2). The abundance of “Hard Coral” and “Soft Coral” estimated by the machine showed errors between 1% (Atlantic) and 4% (Australia), and 0.5% (Indian Ocean) and 3% (Australia), respectively. The remaining groups (Other and Other Invertebrates) showed a consistent error below 2% (Fig. 2).

**Fig. 3:**
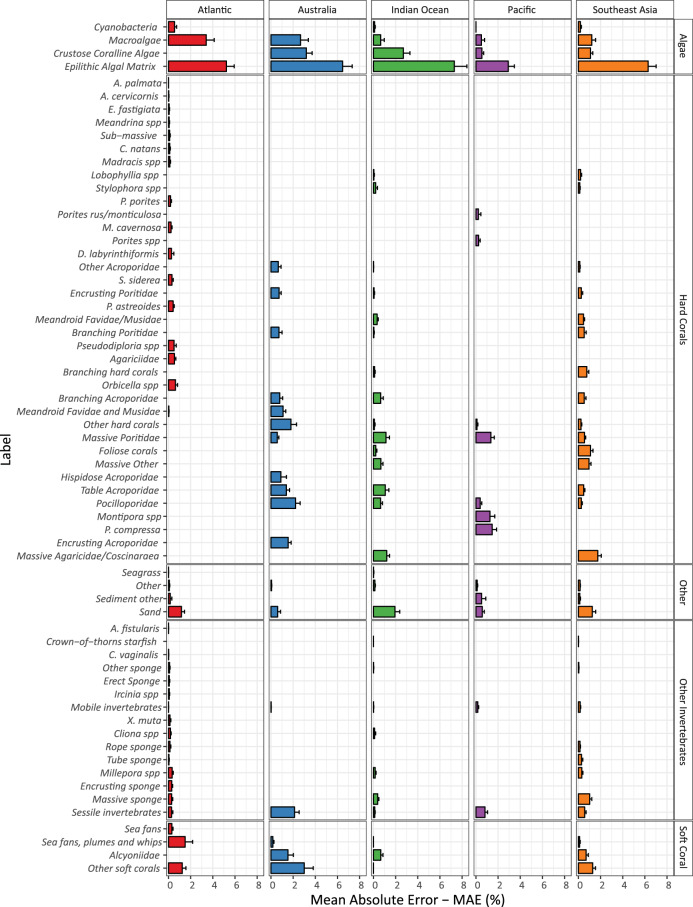
Mean Absolute Error for the automated estimation of the abundance (cover) of key benthic categories. Errors are aggregated by main benthic groups, along the y-axis, and regions, along the x-axis. Solid and error bars represent the mean and 95% Confidence Intervals of the error, respectively. Figure originally published as Fig. 4 in González-Rivero *et al*.^23^.

The error of machine estimates was more consistent within main groups, with the only exception of the “Algae” group (Fig. 3). “Epilithic Algal Matrix”, as a functional group, comprised of a diverse number of algae groups (e.g., turf, cyanobacteria) was the most variable label (5%–7% error). The error of estimations, within the “Hard Coral” group, remained below 2% among regions, while “Soft Coral”, in particular “Other soft corals”, showed an error of up to 3%. This label is comprised of a large diversity of genera and growth forms, while more taxonomically defined labels showed an error below 2%. The remaining labels within the “Other” group, comprised mainly by substrate categories (e.g., sand, terrigenous sediment), and “Other Invertebrates”, comprise of benthic invertebrates other than hard and soft corals, showed a consistently low error (below 1%–2%; Fig. 3).

In addition, the overall performance of automated image annotation was evaluated by 1) correlating the estimates of abundance (cover) produced by the machine against those produced by the observer, and 2) evaluating the overall agreement between machine and observer estimations using the Bland-Altman plot^48^, also called difference plots. Correlation was evaluated using the coefficient of determination (R^2^) from a linear regression model, which also evaluated the significance of this correlation. The R^2^ provides an indication of the intensity of the correlation by evaluating the co-variance between the observer and machine estimations. The Bland-Altman plot determines differences between the two estimations against the observer estimations, or reference *sensu* Krouwer^49^. We used the Bland-Altman plot analysis^50^ to evaluate: (1) the mean of the difference or bias of machine estimations, (2) the homogeneity of the difference between machine and observer across the mean (i.e., over-dispersion) and, (3) the critical difference or agreement limits. The latter refers to the range, within the 95% Confidence Interval, of the difference between the two methods, and can be used as a reference to define where the measurements fall out of the range of the agreement (i.e., precision of the agreement). Bias refers to the difference between the two methods and the Bland-Altman plot can help visualise whether this bias changes across the mean of values evaluated, and therefore a measurement of the consistency of the bias^50^.

**Fig. 4:**
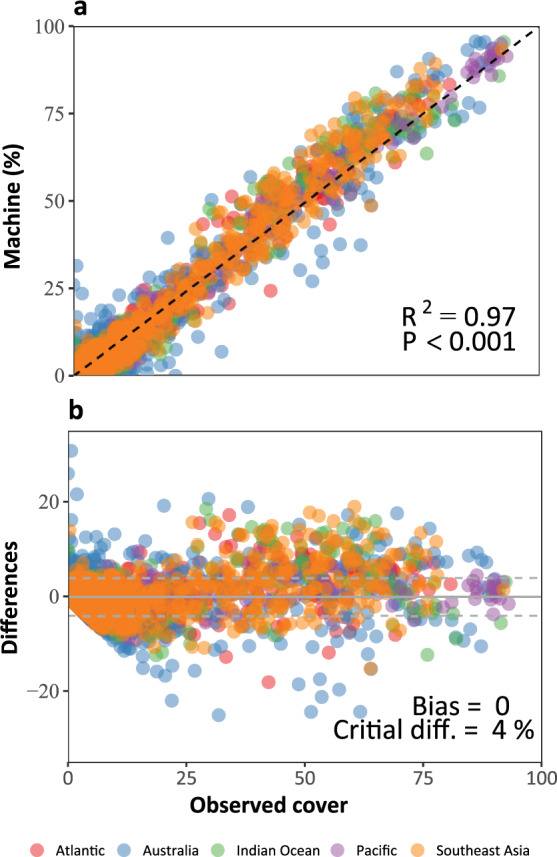
Overall agreement between observer (manual) and machine (networks) estimations of abundance (cover). Agreement is here discretised in two metrics: (**a**) Correlation between machine and observer annotations, and (**b**) bias (Bland-Altman plot). Each filled circle in these panels represents the estimated cover for each label classified by both, the machine and the observer in a given transect. The correlation shows that estimations of benthic abundance by expert observations are significantly represented by the automated estimations (R^2^ = 0.97). The Bland-Altman plot shows that overall the differences (Bias) between machine and observer tend to mean of zero (grey continuous line), and a homogenous error around the mean, defined by Critical Difference (Critical diff.) or the 95% Confidence Interval of the difference between observers and machines (dashed grey lines). Figure originally published in González-Rivero *et al*.^23^ (as Figure A1).

Expectedly, network estimations (machine) of benthic cover were highly correlated with observer estimations for all five global regions (R^2^ = 0.97, P < 0.001, Fig. 4). Most importantly, the differences between the machine and observer were unbiased across the spectrum of benthic cover (mean ~ 0), and the variability around the mean difference was estimated at 4% (Critical Difference or 95% Confidence Interval of the difference) for all labels across the surveyed regions (Fig. 4).

Lastly, to evaluate the machine performance for estimating community structure, pair-wise comparisons of manual and automated estimations of benthic composition within each test transect were executed using the Bray-Curtis similarity index. This index is sensitive to misrepresentation in the automated estimation of abundance for specific labels or benthic groups when compared against manual observations. Therefore, index values of 100% will represent a complete resemblance between machine and observer estimations for community composition. While the Absolute Error already provides a metric for label-specific performance of automated annotations, the community-wide analysis lays out a synthesis analysis to understand how closely represented is the automated estimation of benthic composition against manual observations across the range of communities within a region.

**Fig. 5:**
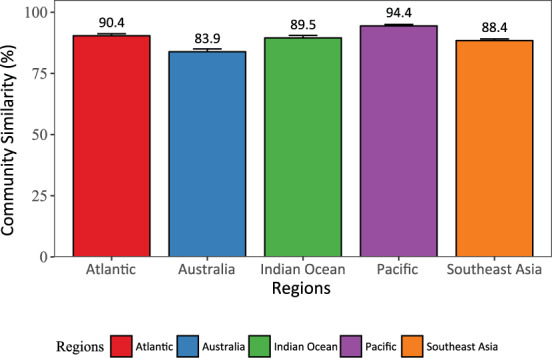
Comparison of compositional similarity within regions. Community similarity between observed and estimated benthic composition per region was evaluated by the Bray-Curtis similarity index. Solid and error bars represent the mean and 95% Confidence Intervals of the error, respectively. Figure originally published in González-Rivero *et al*.^23^ (as Fig. 5).

The comparison of communities within regions using Bray-Curtis similarity index showed the estimations of benthic composition were consistent between observer and machine (i.e., between 84% and 94% of similarity) among and within regions (Fig. 5). Across regions, Australia and South East Asia exhibited the lowest values of similarities, 84% and 88% respectively. Indian Ocean and Atlantic showed similarities around 90%, while Central Pacific Ocean presented up to 94% of similarity between automated estimations and manual observations. Irrespective of the differences in community structure among and within regions, this comparison corroborates the reliability of the machine estimations at both label and community level.

Machine estimations were generally better for well-defined and large organisms in quadrats, such as corals and soft corals, showing the lowest error (<1%–2%). The window size (224 × 224 pixels), however, penalises the estimation of smaller, patchy or less defined organisms, such as algal categories (error 3%–6%). Additional caveats and discussions in terms of the errors and accuracy of data derived from automated classifiers can be found in Beijbom *et al*.^18,19^, González-Rivero *et al*.^23,25^ and Williams *et al*.^22^.

In summary, the validation exercise demonstrates that the trained networks generated cover estimates unbiased compared to those derived from observers, with an overall error of 4%. Thus, our data set represents a useful source of information to support spatio-temporal analysis of coral reefs ranging from small- (presence/absence within quadrats 1 m^2^) to broad-scales (regional/global analysis).

## Usage Notes

Users can identify transects of relevance using a map or file naming convention present on the data repository^34^. The survey ID (“surveyid” in tables) is the unique identifier of each reef location in space and time that users will require as a key cross-reference to navigate within the repository and link the different cover tables, as well as cover records with the imagery folders.

Our relational dataset contains some tables with thousands to millions of rows (e.g., cover, quadrat and annotation tables), hence it is advised to use a software that can handle this structure, such as R, PostGRES, Microsoft Access, etc. Manipulating these data with Microsoft Excel is not advised. For users interested in working with the whole dataset, the best approach will be to create a database, for instance in the MySQL system, and import all the csv files provided within the “tabular-data.zip”. Then the user can relate the tables using the “surveyid”, “imageid” and “quadratid” fields, which are cross-referenced between tables (see Data Records section and Table 3).

### Accessing benthic covers

We provide guidelines to access cover records from the Indian Ocean Region (hereafter called “IOR”) per survey and quadrat as a transferable example for retrieving any cover record. The first step is to download the “tabular-data.zip” file from the repository^34^. As soon as the file is unzipped, 18 csv files (i.e., tables) are extracted including six with cover records, one file for each of the five regions and one summary file. Within the latter, the “seaviewsurvey_surveys.csv” file, the user can directly access the percent cover of main benthic groups (hard coral, algae, soft coral, other invertebrate, and other) summarised per survey (“surveyid” column). Filtering this table by the “ocean” column, users can select the surveys associated only with IOR, in this case 92 surveys. In addition, users can filter the surveys per country (“country” column), link each survey to the zip folder with the images (“folder_name” column) and the geographic location (columns with latitude and longitude at the start and end of the survey).

If users require percentage covers of detailed benthic categories per quadrat they must work with the file “seaviewsurvey_reefcover_indianocean.csv” (if the user is interested in other region/country, please refer to Table 1 to find the appropriate file). Users can relate cover records at the level of surveys (seaviewsurvey_surveys.csv file) with records at the level of quadrats (seaviewsurvey_reefcover_indianocean.csv) using the “surveyid” field.

To summarise percentage covers at the level of quadrats, users must sum the values of the benthic categories of interest. In our example with the IOR, by selecting and merging from the 45 field names of the columns in the “seaviewsurvey_reefcover_indianocean.csv” file. For this purpose, we recommend using the label-set table “seaviewsurvey_labelsets.csv” to understand the labels of different benthic categories, review the descriptions and examples, and guide your merging scheme. Filtering the label-set table by the “region” column, in this case selecting Indian Ocean, users will obtain the 45 categories (under the “label” column) associated with this region. Please note we also provide a reduced set of categories (under “merged_label” and “merged_name” columns) used in the technical validation, however users can merge the categories based on their criteria. A good consistency check to use after merging categories is that the cover fields in each row should always sum to 1.0 (these values are proportions, and 1.0 represents 100%).

To create summaries of the cover data for spatial analyses (at different scales) users will need to consider the cases where from a single georeferenced image there is more than one 1 m^2^ photo-quadrat extracted (see Post-processing of images section). This implies all the quadrats originated from the same image have the same georeference. For this situation, we recommend to aggregate the cover table (in this case seaviewsurvey_reefcover_indianocean.csv) by the “imageid” field, taking the mean cover value among all records (quadrats) for an image. From this point, users can spatially aggregate the images according with their own criteria and/or purposes. For instance, we applied hierarchical clustering to obtain the 30 m test transects for the technical validation. Further details about this method are provided in González-Rivero *et al*.^23^ (see SM4 in Supplementary Material) and Vercelloni *et al*.^37^. When aggregating, users can relate cover records using the “surveyid”, “imageid” and “quadratid” fields. It is important to remind here that the dataset has a unique ID for each survey (five digits), image (nine digits), and quadrat (eleven digits) that link the different tables and files. Thus, in the ID 36001004202 from the IOR cover dataset, the first five digits correspond to the survey ID (36001), this survey ID and the next four digits to the image ID (360010042), and the image ID along with the last two digits to the quadrat ID (36001004202, 02 implies there are at least two quadrats available for the 360010042 image).

### Accessing temporal data

Table 1 identifies which regions/countries have temporal data. Using the “seaviewsurvey_surveys.csv” table users can identify which reef locations have been surveyed multiple times by filtering by the “transectid” field. A transect ID will appear more than once in this field if the transect has been surveyed temporally in more than one year. For instance, filtering the table by Indian Ocean/Maldives, and then filtering the first transect ID, 37001, you will find there are two surveys ID associated with this transect, 37001 and 46001, which were done in 2015 and 2017 respectively as recorded in the “surveydate” field. This means this reef location has available images and cover data for these years. Once the user has identified and selected reef locations with temporal data, we recommend plotting the georeferenced data of the images of the surveys of interest in order to visually explore the spatial overlap between them over the different years and design appropriate temporal comparisons. Further details about temporal assessments are provided in González-Rivero *et al*.^23^ (see SM4 in Supplementary Material), Kennedy *et al*.^39^ and Vercelloni *et al*.^37^.

### Accessing the imagery associated with benthic covers

To inspect the image quadrats associated with the data in detail, users only need the survey ID(s) and to download the set of quadrats required from the “photo-quadrats” directory at the repository^34^. Within this directory, the different zip folders of each survey can be identified with a code (e.g., IND_MDV_37001_201503) that includes the five digits of the survey ID in the middle of the code (i.e., 37001). Alternatively, users can find the zip folder’s name associated with any survey using the “seaviewsurvey_surveys.csv” table and looking at the “folder_name” column. Once a survey has been downloaded and unzipped, all the associated quadrats (jpeg format) are identified with a unique ID, the “quadratid” in which the first five digits correspond to the survey ID. Users can always related each photo-quadrat to the cover records from the data tables using the “quadratid”.

The sets of three raw photographs (downward, left and right facing), in CR2 format (Canon Raw version 2 format) that constitute each survey, including the downward facing files used to generate the quadrats of this dataset will be also accessible through an open-access data repository in the future.

### Accessing annotations data and their images

Extracting the “tabular-data.zip” file users can access nine files with the records of manual annotations used for training and validating each network (one per network). Table 2 lists the networks and the name of the corresponding csv file. For instance using the “annotations_IND_MDV.csv” table and filtering by the “data_set” field users can differentiate if the record was used for either training (“train”) or validation (“test”). The image quadrats specifically associated with the annotations can be accessed from the “annotated-images” directory on the repository^34^. Within this directory, the different zip folders of the networks are identified with the corresponding annotations file name but excluding the “annotations” part. Thus, for the “annotations_IND_MDV.csv” set of annotations, the matching images folder is “IND_MDV.zip”. By associating annotations data and their images with the labelset information (seaviewsurvey_labelsets.csv file), users can extract specific visual examples of the labels used during the manual annotations for the different regions.

Additionally, if users are interested in visual examples of the labels, the Supplementary Table 1 provides the URLs to view multiple examples of images annotated at CoralNet (https://coralnet.ucsd.edu/) for each label. Users need to search for the region and label of interest within the table, click on the relevant URL or also copy the URL and paste it into any browser address bar to access the images directly.

In addition to the manual annotations, within the “tabular-data.zip” file users can have access to one file with all raw records of the automated annotations (“seaviewsurvey_annotations.csv”). The records of this table can be related to the cover records (processed data of covers files) by the “quadratid” fields.

## Supplementary information

Supplementary File 1

## Data Availability

The documented code and examples to implement the automated image analyses method from this study can be found in the following repository: https://github.com/mgonzalezrivero/reef_learning.

## References

[1] Hoegh-Guldberg, O. *et al*. Coral Reefs Under Rapid Climate Change and Ocean Acidification. *Science* 318, 1737-1742, 10.1126/science.1152509%J (2007). 10.1126/science.1152509 18079392

[2] Pandolfi, J. M. *et al*. Global Trajectories of the Long-Term Decline of Coral Reef Ecosystems. *Science* **301**, 955-958, 10.1126/science.1085706%J (2003). 10.1126/science.1085706 12920296

[3] Hughes TP, Graham NA, Jackson JB, Mumby PJ, Steneck RS (2010). Rising to the challenge of sustaining coral reef resilience. Trends in ecology & evolution.

[4] Norström AV (2016). Guiding coral reef futures in the Anthropocene. Frontiers in Ecology and the Environment.

[5] Williams GJ (2019). Coral reef ecology in the Anthropocene. Funct Ecol.

[6] Bellwood DR (2019). Coral reef conservation in the Anthropocene: Confronting spatial mismatches and prioritizing functions. Biological Conservation.

[7] Darling ES (2019). Social–environmental drivers inform strategic management of coral reefs in the Anthropocene. Nature Ecology & Evolution.

[8] Hoegh-Guldberg O, Kennedy EV, Beyer HL, McClennen C, Possingham HP (2018). Securing a Long-term Future for Coral Reefs. Trends in Ecology & Evolution.

[9] McCook LJ (2010). Adaptive management of the Great Barrier Reef: a globally significant demonstration of the benefits of networks of marine reserves. Proceedings of the National Academy of Sciences.

[10] Barnes ML (2019). Social-ecological alignment and ecological conditions in coral reefs. Nature Communications.

[11] Beyer HL (2018). Risk-sensitive planning for conserving coral reefs under rapid climate change. Conservation Letters.

[12] McLeod E (2019). The future of resilience-based management in coral reef ecosystems. Journal of Environmental Management.

[13] van Oppen MJH (2017). Shifting paradigms in restoration of the world’s coral reefs. Global Change Biology.

[14] Pendleton, L. H. *et al*. Disrupting data sharing for a healthier ocean. *ICES Journal of Marine Science* **76**, 1415–1423, 10.1093/icesjms/fsz068%J (2019).

[15] Costello MJ, Michener WK, Gahegan M, Zhang Z-Q, Bourne PE (2013). Biodiversity data should be published, cited, and peer reviewed. Trends in Ecology & Evolution.

[16] Obura, D. O. *et al*. Coral Reef Monitoring, Reef Assessment Technologies, and Ecosystem-Based Management. **6**, 10.3389/fmars.2019.00580 (2019).

[17] Hughes TP (2017). Coral reefs in the Anthropocene. Nature.

[18] Beijbom O (2015). Towards Automated Annotation of Benthic Survey Images: Variability of Human Experts and Operational Modes of Automation. PloS one.

[19] Beijbom O (2016). Improving automated annotation of benthic survey images using wide-band fluorescence. Scientific reports.

[20] Hedley, D. J. *et al*. Remote Sensing of Coral Reefs for Monitoring and Management: A Review. *Remote Sensing* **8**, 10.3390/rs8020118 (2016).

[21] Madin EMP, Darling ES, Hardt MJ (2019). Emerging Technologies and Coral Reef Conservation: Opportunities, Challenges, and Moving Forward. Frontiers in Marine Science.

[22] Williams, I. D. *et al*. Leveraging Automated Image Analysis Tools to Transform Our Capacity to Assess Status and Trends of Coral Reefs. *Frontiers in Marine Science* **6**, 10.3389/fmars.2019.00222 (2019).

[23] González-Rivero M (2020). Monitoring of Coral Reefs Using Artificial Intelligence: A Feasible and Cost-Effective Approach. Remote Sensing.

[24] Gouezo M (2019). Drivers of recovery and reassembly of coral reef communities. Proc Biol Sci.

[25] González-Rivero M (2016). Scaling up Ecological Measurements of Coral Reefs Using Semi-Automated Field Image Collection and Analysis. Remote Sensing.

[26] Obura DO (2019). Coral Reef Monitoring, Reef Assessment Technologies, and Ecosystem-Based Management. Frontiers in Marine Science.

[27] Bruno JF, Sweatman H, Precht WF, Selig ER, Schutte VGW (2009). Assessing evidence of phase shifts from coral to macroalgal dominance on coral reefs. Ecology.

[28] Selig ER, Bruno JF (2010). A Global Analysis of the Effectiveness of Marine Protected Areas in Preventing Coral Loss. PLOS ONE.

[29] Bruno JF, Selig ER (2007). Regional Decline of Coral Cover in the Indo-Pacific: Timing, Extent, and Subregional Comparisons. PLOS ONE.

[30] Côté IM, Gill JA, Gardner TA, Watkinson AR (2005). Measuring coral reef decline through meta-analyses. Philos Trans R Soc Lond B Biol Sci.

[31] Jackson, J., Donovan, M., Cramer, K. & Lam, V. Status and trends of Caribbean coral reefs: 1970–2012. (Global Coral Reef Monitoring Network, 2014).

[32] Moritz, C. *et al*. Status and Trends of Coral Reefs of the Pacific. (Global Coral Monitoring Network, 2018).

[33] Obura, D. O. *et al*. Coral Reef Status Report for the Western Indian Ocean. (Global Coral Reef Monitoring Network, 2017).

[34] González-Rivero J (2019). University of Queensland Library.

[35] González-Rivero M (2014). The Catlin Seaview Survey - kilometre-scale seascape assessment, and monitoring of coral reef ecosystems. Aquatic Conservation: Marine and Freshwater Ecosystems.

[36] Peterson, E. E. *et al*. Monitoring through many eyes: Integrating scientific and crowd-sourced datasets to improve monitoring of the Great Barrier Reef. *arXiv preprint arXiv:1808.05298* (2018).

[37] Vercelloni, J. *et al*. Forecasting intensifying disturbance effects on coral reefs. *Global Change Biology*, 10.1111/gcb.15059 (2020). 10.1111/gcb.15059 32115808

[38] Bryant DEP (2017). Comparison of two photographic methodologies for collecting and analyzing the condition of coral reef ecosystems. Ecosphere.

[39] Kennedy EV (2020). Coral Reef Community Changes in Karimunjawa National Park, Indonesia: Assessing the Efficacy of Management in the Face of Local and Global Stressors. Journal of Marine Science and Engineering.

[40] Althaus F (2015). A Standardised Vocabulary for Identifying Benthic Biota and Substrata from Underwater Imagery: The CATAMI Classification Scheme. PLOS ONE.

[41] Wallace, C. C. *Staghorn corals of the world: a revision of the coral genus Acropora (Scleractinia; Astrocoeniina; Acroporidae) worldwide, with emphasis on morphology, phylogeny and biogeography / Carden C. Wallace*. (CSIRO Publishing, 1999).

[42] Simonyan, K. & Zisserman, A. Very deep convolutional networks for large-scale image recognition. *arXiv preprint arXiv:1409.1556* (2014).

[43] Beijbom, O., Edmunds, P. J., Kline, D. I., Mitchell, B. G. & Kriegman, D. In *2012 IEEE Conference on Computer Vision and Pattern Recognition*. 1170–1177 (IEEE).

[44] Brown EK (2004). Development of benthic sampling methods for the Coral Reef Assessment and Monitoring Program (CRAMP) in Hawai’i. Pacific Science.

[45] Murdoch, T. J. Status and Trends of Bermuda Reefs and Fishes: 2015 Report Card. (Bermuda Zoological Society, Flatts, Bermuda, 2017).

[46] Sweatman, H. H. *et al*. Long-term monitoring of the Great Barrier Reef. Status Report Number 7. (Australian Institute of Marine Science, 2005).

[47] Ninio R, Delean J, Osborne K, Sweatman H (2003). Estimating cover of benthic organisms from underwater video images: variability associated with multiple observers. Marine Ecology-Progress Series.

[48] Bland JM, Altman DG (1999). Measuring agreement in method comparison studies. Statistical Methods in Medical Research.

[49] Krouwer JS (2008). Why Bland–Altman plots should use X, not (Y+X)/2 when X is a reference method. Statistics in Medicine.

[50] Giavarina D (2015). Understanding Bland Altman analysis. Biochemia Medica.

