# Supplementary material for: A contemporary baseline record of the world’s coral reefs
Source: Sci Data. 2020 Oct 20;7:355. doi: 10.1038/s41597-020-00698-6 (PMC7576589; doi:10.1038/s41597-020-00698-6)
Supplement: Supplementary file 1 [file 41597_2020_698_MOESM1_ESM.pdf]

Supplementary Information

A contemporary baseline record of the world's coral reefs

Alberto Rodriguez-Ramirez[1,2]*, Manuel González-Rivero[1,3,4]*, Oscar Beijbom[1,5], Christophe Bailhache[6], Pim Bongaerts[1,7], Kristen T. Brown[2,4], Dominic E.P. Bryant[4], Peter Dalton[1], Sophie Dove[2,4], Anjani Ganase[4], Emma V. Kennedy[1,8], Catherine J.S. Kim[4], Sebastian Lopez-Marcano[1,9], Benjamin P. Neal[1,10], Veronica Z. Radice[4], Julie Vercelloni[1,4,11], Hawthorne L. Beyer[2,4] and Ove Hoegh-Guldberg[1,2,4]*

[1] Global Change Institute, The University of Queensland, 4072 St Lucia, QLD, Australia
[2] School of Biological Sciences, The University of Queensland, 4072 St Lucia. QLD, Australia
[3] Australian Institute of Marine Science, 4810 Cape Cleveland, QLD, Australia
[4] Australian Research Council (ARC) Centre for Excellence for Coral Reef Studies at The University of Queensland, 4072 St Lucia. QLD, Australia
[5] Berkeley Artificial Intelligence Research, University of California, Berkeley, CA 94720, USA
[6] Underwater Earth, Sydney, NSW, Australia
[7] California Academy of Sciences, San Francisco, CA 94118, USA
[8] School of Earth and Environmental Sciences, Faculty of Science, The University of Queensland, 4072, Qld, Australia
[9] Australian Rivers Institute, Griffith University, Gold Coast Campus, Australia
[10] Colby College, Environmental Studies Department, Waterville, Maine (ME), USA 04901 and Bigelow Laboratory for Ocean Sciences, East Boothbay, ME 04544, USA
[11] ARC Centre of Mathematical and Statistical Frontiers, Queensland University of Technology, and School of Mathematical Sciences, Science and Engineering Faculty, Queensland University of Technology, 4000 Brisbane, QLD, Australia

*These authors contributed equally to this work.
Corresponding authors: Alberto Rodriguez-Ramirez (alberto.rodriguezl@uqconnect.edu.au), Manuel González-Rivero (m.gonzalezrivero@aims.gov.au, Ove Hoegh-Guldberg (oveh@uq.edu.au).

Table of contents

	Page
Supplementary Figure 1. The "SVII", an underwater propulsion vehicle customised with high-definition cameras used to survey coral reefs. .	2
Supplementary Figure 2. Reef locations (surveys) mapped by coral reef region.	2
Supplementary Figure 3. Hierarchical structure of the photographic surveys and dataset.	3
Supplementary Table 1. URLs for accessing visual examples of the labels used during the training.	4-9
References	9

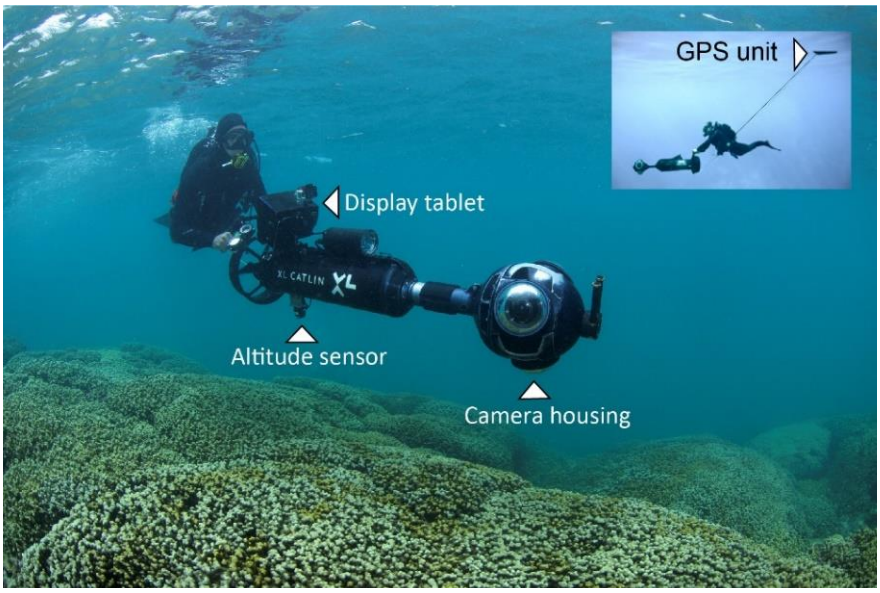

Supplementary Figure 1. The "SVII", an underwater propulsion vehicle customised with high-definition cameras used to survey coral reefs. This SVII camera system was invented by Underwater Earth and customised for scientific purposes by the Global Change Institute at the University of Queensland. The inset illustrates how a GPS unit was tethered to the surface for recording geographic positions and geo-referencing each image. A detailed description of the methodological aspects used during the field surveys has been published in González-Rivero *et al.*[1-3]

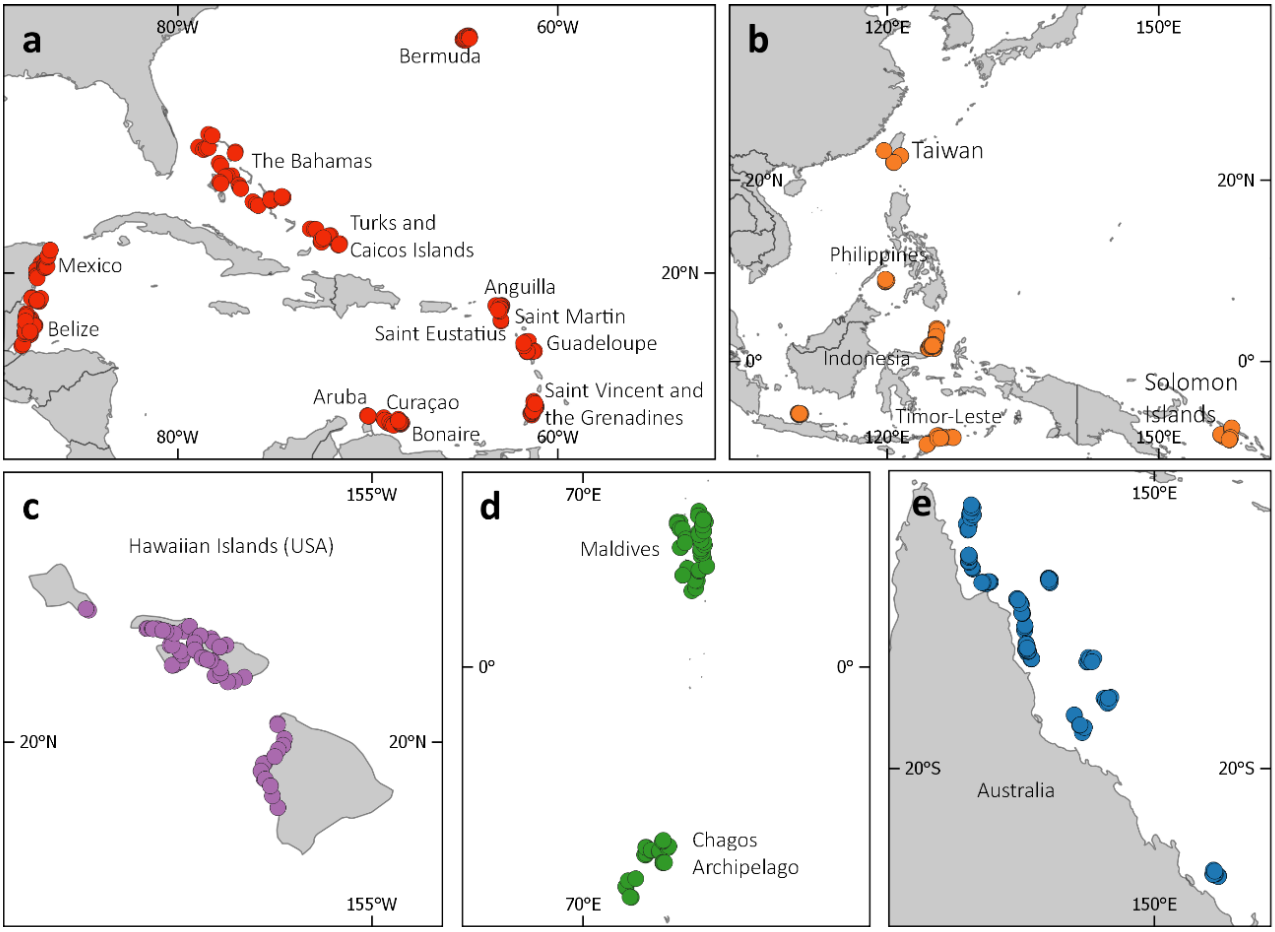

Supplementary Figure 2. Reef locations (surveys) mapped by coral reef region. 860 surveys around the world, across 23 countries or territories from (a) the Western Atlantic Ocean, (b) Southeast Asia, (c) Central Pacific Ocean, (d) Central Indian Ocean, (e) and Eastern Australia, were performed from 2012-2018. Reef locations are represented by dots colour-coded according to the survey region in Fig 1 and Table 1. QGIS software was used to generate the maps using the layer "Countries WGS84" downloaded from ArcGIS Hub (http://hub.arcgis.com/datasets/UIA::countries-wgs84).

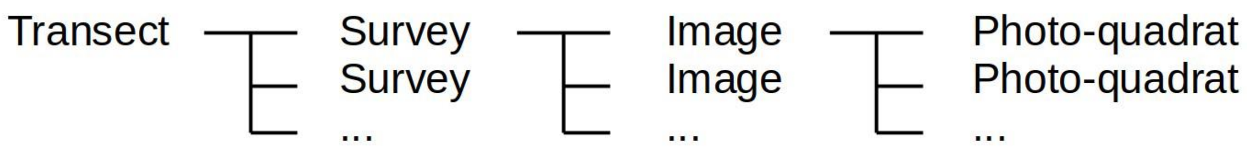

Supplementary Figure 3. Hierarchical structure of the photographic surveys and dataset. A transect refers to a reef location that can be surveyed multiple times (e.g. in different years). Thus, one transect might have multiple surveys. Each survey involves the collection of many images. From each image one or more standardised photo-quadrat images can be extracted.

Supplementary Table 1. URLs for accessing visual examples of the labels used during the training.

Region	Label code	Label description and/or examples	URL
Atlantic	AARC	Tube sponges: *Aplysina archeri*	https://coralnet.ucsd.edu/label/406/
Atlantic	ACER	*Acropora cervicornis*	https://coralnet.ucsd.edu/label/376/
Atlantic	ACOM	Sponges: Upright narrow tubes branches arborescent e.g. *A. compressa*	https://coralnet.ucsd.edu/label/415/
Atlantic	Acra	Massive sponges: *Aiolochroia crassa*	https://coralnet.ucsd.edu/label/871/
Atlantic	AFIS	Sponges: *Aplysina fistularis*	https://coralnet.ucsd.edu/label/407/
Atlantic	AGAR	Agariciidae. Plates and encrusting corals of the genera *Agaricia, Undaria* and *Helioseris*	https://coralnet.ucsd.edu/label/377/
Atlantic	AMAT	Multi-specific assemblages of filamentous algae and CCA; 1 cm or less in height	https://coralnet.ucsd.edu/label/404/
Atlantic	APAL	*Acropora palmata*	https://coralnet.ucsd.edu/label/223/
Atlantic	ApFuCa	Rope sponges; Height >> basal area; Spread along the substrate. e.g. *Aplysina fulva, A. cauliformis*	https://coralnet.ucsd.edu/label/1258/
Atlantic	ATUB	Tube sponges: *Agelas tubulata*	https://coralnet.ucsd.edu/label/414/
Atlantic	CCA	Crustose coralline algae	https://coralnet.ucsd.edu/label/101/
Atlantic	CDEL	*Cliona delitrix*	https://coralnet.ucsd.edu/label/411/
Atlantic	CNAT	*Colpophyllia natans*	https://coralnet.ucsd.edu/label/378/
Atlantic	CPLI	Tube sponges: *Callyspongia plicifera*	https://coralnet.ucsd.edu/label/413/
Atlantic	CVAG	*Callyspongia vaginalis*	https://coralnet.ucsd.edu/label/412/
Atlantic	CVIR	*Cliona viridis* complex	https://coralnet.ucsd.edu/label/410/
Atlantic	CYAN	Cyanobacteria	https://coralnet.ucsd.edu/label/143/
Atlantic	Dict	Macroalgae: *Dictyota* sp.	https://coralnet.ucsd.edu/label/401/
Atlantic	DLAB	*Diploria labyrinthiformis*	https://coralnet.ucsd.edu/label/381/
Atlantic	DLAB-BL	Bleached *Diploria labyrinthiformis*	https://coralnet.ucsd.edu/label/697/
Atlantic	EFAS	*Eusmilia fastigiata*	https://coralnet.ucsd.edu/label/382/
Atlantic	ENGR1	Encrusting soft corals	https://coralnet.ucsd.edu/label/400/
Atlantic	ENSP	Encrusting sponges: < 5 cm height (for whole individual); e.g. *S. ruetzleri*; *Haliclona* sp.	https://coralnet.ucsd.edu/label/147/
Atlantic	ERGR	Erect soft corals	https://coralnet.ucsd.edu/label/416/
Atlantic	ERHD	Macroalgae: filamentous red algae	https://coralnet.ucsd.edu/label/696/
Atlantic	ERSP	Erect sponges: Upright narrow tubes branches arborescent; >1 cm height; height >>basal area	https://coralnet.ucsd.edu/label/148/
Atlantic	FISH	Fish	https://coralnet.ucsd.edu/label/460/
Atlantic	Gnep	*Geodia neptuni*	https://coralnet.ucsd.edu/label/405/
Atlantic	GORG	Sea fans; e.g *Gorgonia* sp.	https://coralnet.ucsd.edu/label/178/
Atlantic	Hali	Calcifying macroalgae: *Halimeda* sp.	https://coralnet.ucsd.edu/label/403/
Atlantic	IRCI	*Ircinia* sp.	https://coralnet.ucsd.edu/label/408/
Atlantic	Lvar	Macroalgae: *Lobophora* sp.	https://coralnet.ucsd.edu/label/402/
Atlantic	MADR	*Madracis* sp.	https://coralnet.ucsd.edu/label/387/
Atlantic	MCAV	*Montastraea cavernosa*	https://coralnet.ucsd.edu/label/110/
Atlantic	MEA-HC	Meandroid corals including *Dendrogyra*; *Isophyllia*; *Manicina*; *Mycetophyllia*; *Mussa*; *Scolymia*	https://coralnet.ucsd.edu/label/2116/
Atlantic	MEAN	*Meandrina* sp.	https://coralnet.ucsd.edu/label/388/
Atlantic	Mille	*Millepora* sp.	https://coralnet.ucsd.edu/label/79/
Atlantic	Mille-BL	Bleached *Millepora*	https://coralnet.ucsd.edu/label/459/
Atlantic	MALG	Macroalgae: Upright macroalgae > 1 cm in height (all genera and species)	https://coralnet.ucsd.edu/label/181/
Atlantic	MOB-INV	Other mobile invertebrates: i.e Echinoderms; lobster	https://coralnet.ucsd.edu/label/180/
Atlantic	MSE-HC	Sub-massive corlas including *Dichocoenia*; *Favia*; *Solenastrea*; *Stephanocoenia*	https://coralnet.ucsd.edu/label/2113/
Atlantic	MTUN	Other Invertebrates: Mat tunicate (ascidians)	https://coralnet.ucsd.edu/label/462/

Region	Label code	Label description and/or examples	URL
Atlantic	Ndig	Tube sponges: *Niphates digitalis*	https://coralnet.ucsd.edu/label/565/
Atlantic	OCOM	*Orcbicella* complex: *O. annularis*; O. *faveolata*; *O. franksi*	https://coralnet.ucsd.edu/label/392/
Atlantic	OCOM-BL	Bleached *Orcbicella* complex	https://coralnet.ucsd.edu/label/457/
Atlantic	OTH-SINV	Other sesile invertebrates: bryozoa; clams; tunicates (ascidians); soft hexacorrallia; hydroids	https://coralnet.ucsd.edu/label/179/
Atlantic	PASTR	*Porites astreoides*	https://coralnet.ucsd.edu/label/393/
Atlantic	PLAK	Massive sponges: *Plakortis sp.*	https://coralnet.ucsd.edu/label/817/
Atlantic	PPOR	Porites branching: *P. porites*; *P. divaricata*; *P. furcata*	https://coralnet.ucsd.edu/label/394/
Atlantic	PSEU	*Pseudodiploria* sp.	https://coralnet.ucsd.edu/label/395/
Atlantic	PSEU-BL	Bleached *Pseudodiploria* sp.	https://coralnet.ucsd.edu/label/458/
Atlantic	ROSP	Rope sponges; Height >> basal area; Spread along the substrate. e.g. *S. aurea*	https://coralnet.ucsd.edu/label/1191/
Atlantic	Sand	Sand	https://coralnet.ucsd.edu/label/84/
Atlantic	SCplu	Soft coral plumes and wipes	https://coralnet.ucsd.edu/label/556/
Atlantic	Seagrass	Seagrass	https://coralnet.ucsd.edu/label/94/
Atlantic	Sediment	Terrigenous sediments	https://coralnet.ucsd.edu/label/95/
Atlantic	SpMass	Massive sponges: Large basal area to body size and height > 5 cm	https://coralnet.ucsd.edu/label/183/
Atlantic	Sponge	Other sponges	https://coralnet.ucsd.edu/label/102/
Atlantic	SPvase	Vase sponges: vase and small barrel group (tube opening ~ height; basal area < opening)	https://coralnet.ucsd.edu/label/795/
Atlantic	SSID	*Siderastrea siderea*	https://coralnet.ucsd.edu/label/111/
Atlantic	Turf	Turf algae: Multi-specific assemblages of filamentous algae ; 1 cm or less in height	https://coralnet.ucsd.edu/label/82/
Atlantic	Turfsa	Terrigenous sediments mixed with algal turf (mostly sediments)	https://coralnet.ucsd.edu/label/463/
Atlantic	Unc	Unclear	https://coralnet.ucsd.edu/label/118/
Atlantic	UTEN	*Undaria tenuifoila*	https://coralnet.ucsd.edu/label/398/
Atlantic	XMUT	*Xestospongia muta*	https://coralnet.ucsd.edu/label/409/
Atlantic	ZOAN	Other Invertebrates: Zoanthid	https://coralnet.ucsd.edu/label/133/
Pacific Hawaii	Blpocill	Bleached *Pocillopora* sp.	https://coralnet.ucsd.edu/label/2490/
Pacific Hawaii	B_Monti	Bleached *Montipora* sp.	https://coralnet.ucsd.edu/label/212/
Pacific Hawaii	CCA	Crustose coralline algae	https://coralnet.ucsd.edu/label/101/
Pacific Hawaii	EAM_DHC	Epilithic algal matrix: Multi-specific algal assemblage smothering reef surface 1 cm or less in height	https://coralnet.ucsd.edu/label/1432/
Pacific Hawaii	FISH	Fish	https://coralnet.ucsd.edu/label/460/
Pacific Hawaii	Lepto	*Leptoseris sp.*	https://coralnet.ucsd.edu/label/67/
Pacific Hawaii	LSUB_SAND	Loose substrate: sand	https://coralnet.ucsd.edu/label/1702/
Pacific Hawaii	LSUB_SEDI	Loose substrate: terrigenous sediments	https://coralnet.ucsd.edu/label/1704/
Pacific Hawaii	LSUB_VOL	Volcanic sediment	https://coralnet.ucsd.edu/label/2576/
Pacific Hawaii	MALGAE	Macrolagae: Upright macroalgae > 1 cm in height (all genera and species)	https://coralnet.ucsd.edu/label/2575/
Pacific Hawaii	MFLAB	*Montipora flabellata*	https://coralnet.ucsd.edu/label/439/
Pacific Hawaii	MINV_Dia	Mobile invertebrates: *Diadema/Ehinothix*	https://coralnet.ucsd.edu/label/2577/
Pacific Hawaii	MINV_Ech	Mobile invertebrates*: Echinometra*	https://coralnet.ucsd.edu/label/2579/
Pacific Hawaii	MINV_Tri	Mobile invertebrates: *Tripneustes*	https://coralnet.ucsd.edu/label/2578/
Pacific Hawaii	MON_Cap_br	*Montipora capitata* morpho 2 branching	https://coralnet.ucsd.edu/label/2581/
Pacific Hawaii	MON_Cap_pl	*Montipora capitata* morpho 1 plating/encrusting	https://coralnet.ucsd.edu/label/2580/
Pacific Hawaii	MPATU	*Montipora patula*	https://coralnet.ucsd.edu/label/440/
Pacific Hawaii	Other	Other	https://coralnet.ucsd.edu/label/103/
Pacific Hawaii	PAV_Due	*Pavona duerdeni*	https://coralnet.ucsd.edu/label/2584/
Pacific Hawaii	PEYDO	*Pocillopora eydouxi*	https://coralnet.ucsd.edu/label/449/

Region	Label code	Label description and/or examples	URL
Pacific Hawaii	POCI_CAU	*Pocillopora meandrina/ligulata*	https://coralnet.ucsd.edu/label/2573/
Pacific Hawaii	POR_Com_fi	*Porties compressa* mopho 1 fingers	https://coralnet.ucsd.edu/label/2570/
Pacific Hawaii	POR_Com_fu	*Porites compressa* morpho 2 fused branches	https://coralnet.ucsd.edu/label/2571/
Pacific Hawaii	POR_EP_B	Bleached Plate-encrusting *Porites* sp.	https://coralnet.ucsd.edu/label/2589/
Pacific Hawaii	POR-MASS	*Porites lobata/lutea*	https://coralnet.ucsd.edu/label/171/
Pacific Hawaii	POR-MASS-B	Bleached *Porites lobata/lutea*	https://coralnet.ucsd.edu/label/2586/
Pacific Hawaii	POR_NOD	*Porites* nodular branches	https://coralnet.ucsd.edu/label/2569/
Pacific Hawaii	POR_OTH_EP	*Porites rus/monticulosa*	https://coralnet.ucsd.edu/label/2572/
Pacific Hawaii	PVAR	*Pavona varians*	https://coralnet.ucsd.edu/label/442/
Pacific Hawaii	SINV_HEX_O	Other sesile invertebrates soft hexacorallia	https://coralnet.ucsd.edu/label/1454/
Pacific Hawaii	SINV_SPO_M	Sponges: Massive or encrusting	https://coralnet.ucsd.edu/label/1405/
Pacific Hawaii	Unc	Unclear	https://coralnet.ucsd.edu/label/118/
Pacific Hawaii	Unk	Unknown	https://coralnet.ucsd.edu/label/1647/
Indian Ocean	BRA_ARB_Ac	Branching *Acropora sp.*	https://coralnet.ucsd.edu/label/1408/
Indian Ocean	BRA_BLC	Bleached branching corals	https://coralnet.ucsd.edu/label/1416/
Indian Ocean	BRA_DIG_Ac	Digitate *Acropora* sp*. e.g. A. humulis*	https://coralnet.ucsd.edu/label/1411/
Indian Ocean	BRA_FIN_Se	Fine branching non-Acroporids: *Seriatopora* sp.	https://coralnet.ucsd.edu/label/1414/
Indian Ocean	BRA_OTH	Other branching corals: *Anacropora/Echinopora* (excluding *Pocillopora and Stylophora)*	https://coralnet.ucsd.edu/label/1415/
Indian Ocean	BRA_RND_St	Branching *Stylophora* sp.	https://coralnet.ucsd.edu/label/1410/
Indian Ocean	BRA_SMO_Po	Branching *Porites* sp.	https://coralnet.ucsd.edu/label/1413/
Indian Ocean	BRA_TAB-Ac	*Acropora* sp.: corymbose/tabular/plate (no digitate)	https://coralnet.ucsd.edu/label/1412/
Indian Ocean	BRA_VER_Po	*Pocillopora* sp.	https://coralnet.ucsd.edu/label/1409/
Indian Ocean	CAL_CCA_DC	Calcifying calcareous crustose algae: smothering reef surface	https://coralnet.ucsd.edu/label/1403/
Indian Ocean	CAL_CCA_RB	Calcifying calcareous crustose algae: smothering rubble	https://coralnet.ucsd.edu/label/1597/
Indian Ocean	CYANO_DHC	Cyanobacteria smothering reef surface	https://coralnet.ucsd.edu/label/1439/
Indian Ocean	EAM_DHC	Epilithic algal matrix: Multi-specific algal assemblage smothering reef surface 1 cm or less in height	https://coralnet.ucsd.edu/label/1432/
Indian Ocean	EAM_RB	Epilithic algal matrix: Multi-specific algal assemblage smothering rubble 1 cm or less in height	https://coralnet.ucsd.edu/label/1433/
Indian Ocean	FISH	Fish	https://coralnet.ucsd.edu/label/460/
Indian Ocean	LSUB_SAND	Loose substrate: sand	https://coralnet.ucsd.edu/label/1702/
Indian Ocean	MACR_Cal_H	Calcifying macroalgae: *Halimeda* sp.	https://coralnet.ucsd.edu/label/1442/
Indian Ocean	MACR_Fil_A	Filamentous macroalgae	https://coralnet.ucsd.edu/label/1448/
Indian Ocean	MACR_Fol_O	Other foliose macroalgae	https://coralnet.ucsd.edu/label/1605/
Indian Ocean	MASE_BLC	Bleached Massive/Submassive/Encrusting corals	https://coralnet.ucsd.edu/label/1424/
Indian Ocean	MASE_LRG_O	Massive Submassive Encrusting colonies with large visible rounded polyps: *Diploastrea; Favia; Favites; Montastraea; Galaxea; Goniopora; Astreopora*	https://coralnet.ucsd.edu/label/1593/
Indian Ocean	MASE_MEA_L	*Lobophyllia* sp.	https://coralnet.ucsd.edu/label/1418/
Indian Ocean	MASE_MEA_O	Massive Submassive Encrusting colonies with meandering ridges and valleys resembling brain: *Platygyra; Leptoria; Goniastrea*	https://coralnet.ucsd.edu/label/1417/
Indian Ocean	MASE_SML_O	Massive Submassive Encrusting colonies with small or invisible polyps (including columnar forms): *Pavona; Psammocora; Coscinaraea; Gardineroseris*	https://coralnet.ucsd.edu/label/1594/
Indian Ocean	MASE_SMO_P	Family Poritidae; massive/sub-massive morphology e.g. *P. lobata; P. lutea*	https://coralnet.ucsd.edu/label/1596/
Indian Ocean	MINV_COTS	Crown-of-thorns starfish	https://coralnet.ucsd.edu/label/1648/
Indian Ocean	MINV_OTH	Other mobile invertebrates: sea cucumbers/sea urchins/sea stars/lobster	https://coralnet.ucsd.edu/label/1455/
Indian Ocean	NON_FREE	Non hermatypic corals: Free living (*Fungia* etc.)	https://coralnet.ucsd.edu/label/1429/
Indian Ocean	NON_MIL	Non Hermatypic: *Millepora*	https://coralnet.ucsd.edu/label/1655/
Indian Ocean	SGRASS	Seagrass	https://coralnet.ucsd.edu/label/1705/

Region	Label code	Label description and/or examples	URL
Indian Ocean	SINV_HEX_O	Other sesile invertebrates soft hexacorallia	https://coralnet.ucsd.edu/label/1454/
Indian Ocean	SINV_HYD	Hydroids feathery types	https://coralnet.ucsd.edu/label/1602/
Indian Ocean	SINV_OTH	Other sesile invertebrates: Bryozoa, clams	https://coralnet.ucsd.edu/label/1437/
Indian Ocean	SINV_SFC_A	Soft coral; Common Alcyoniidae: *Lobophytum; Sarcophyton*	https://coralnet.ucsd.edu/label/1434/
Indian Ocean	SINV_SFC_E	Sea fans/plumes/branching whipes	https://coralnet.ucsd.edu/label/1436/
Indian Ocean	SINV_SFC_O	Other soft-corals no common Alcyoniidae	https://coralnet.ucsd.edu/label/1459/
Indian Ocean	SINV_SPO_C	Sponges: Encrusting *Cliona* sp.	https://coralnet.ucsd.edu/label/2089/
Indian Ocean	SINV_SPO_F	Sponges: Fan shaped forms	https://coralnet.ucsd.edu/label/2092/
Indian Ocean	SINV_SPO_M	Sponges: Massive or encrusting	https://coralnet.ucsd.edu/label/1405/
Indian Ocean	SINV_TUN	Tunicates (ascidians)	https://coralnet.ucsd.edu/label/1603/
Indian Ocean	TFP_RDG_Al	Thin, Foliose, and Plate colonies (excluding *Acropora, Porites*) with/without visible relief structure: e.g. *Echinophyllia; Turbinaria; Montipora; Echinopora*	https://coralnet.ucsd.edu/label/1426/
Indian Ocean	TFP_RND_Al	Thin, Foliose, and Plating (except Acroporids) colonies with visible rounded corallites on the plates: Includes *Turbinaria, Echinopora*	https://coralnet.ucsd.edu/label/1427/
Indian Ocean	TFP_SMO_Po	Family Poritidae; encrusting morphology e.g. *P. lichen*	https://coralnet.ucsd.edu/label/1425/
Indian Ocean	Trans	Transect hardware	https://coralnet.ucsd.edu/label/107/
Indian Ocean	Unc	Unclear	https://coralnet.ucsd.edu/label/118/
Pacific Australia	ACR-BRA	Family Acroporidae; branching morphology (excluding hispidose type branching)	https://coralnet.ucsd.edu/label/168/
Pacific Australia	ACR-HIP	Family Acroporidae; hispidose morphology	https://coralnet.ucsd.edu/label/804/
Pacific Australia	ACR-OTH	Other corals from the family Acroporidae (e.g. *Isopora*)	https://coralnet.ucsd.edu/label/974/
Pacific Australia	ACR-PE	Acroporidae plate/encrusting	https://coralnet.ucsd.edu/label/169/
Pacific Australia	ACR-TCD	Family Acroporidae; table; corymbose and digitate morphologies	https://coralnet.ucsd.edu/label/167/
Pacific Australia	ALC-SF	Soft coral; Common Alcyoniidae: *Lobophytum; Sarcophyton*	https://coralnet.ucsd.edu/label/176/
Pacific Australia	CCA	Crustose coralline algae	https://coralnet.ucsd.edu/label/101/
Pacific Australia	DSUB	Epilithic algal matrix: Multi-specific algal assemblage smothering reef surface 1 cm or less in height	https://coralnet.ucsd.edu/label/182/
Pacific Australia	FAV-MUS	Families Favidae and Mussidae; massive and meandroid morphologies	https://coralnet.ucsd.edu/label/174/
Pacific Australia	GORG	Sea fans/plumes/branching whipes	https://coralnet.ucsd.edu/label/178/
Pacific Australia	MAENR	Macroalgae Encrusting red	https://coralnet.ucsd.edu/label/322/
Pacific Australia	MALG	Macroalgae: Upright macroalgae > 1 cm in height (all genera and species)	https://coralnet.ucsd.edu/label/181/
Pacific Australia	OTH-HC	Other hard coral including all other groups not represented by the other coral categories in Australia	https://coralnet.ucsd.edu/label/175/
Pacific Australia	OTH-SF	Other soft-corals outside of the common Alcyoniidae and erect forms (sea fans plumes whips): e.g. Xeniidae; Nepthtydae; *Tubipora; Briareum; Heliopora*	https://coralnet.ucsd.edu/label/177/
Pacific Australia	OTH-SINV	Other sesile invertebrates: bryozoa; clams; tunicates (ascidians); soft hexacorrallia; hydroids	https://coralnet.ucsd.edu/label/179/
Pacific Australia	POCI	Family Pocilloporidae; all genera and species	https://coralnet.ucsd.edu/label/170/
Pacific Australia	POR-BRA	Family Poritidae; branching morphology e.g. *P. cylindrica*	https://coralnet.ucsd.edu/label/173/
Pacific Australia	POR-ENC	Family Poritidae; encrusting morphology e.g. *P. lichen*	https://coralnet.ucsd.edu/label/172/
Pacific Australia	POR-MASS	Family Poritidae; massive/sub-massive morphology e.g. *P. lobata; P. lutea*	https://coralnet.ucsd.edu/label/171/
Pacific Australia	Sand	Sand	https://coralnet.ucsd.edu/label/84/
Pacific Australia	Turf	Turf algae: Multi-specific assemblages of filamentous algae ; 1 cm or less in height	https://coralnet.ucsd.edu/label/82/
Pacific Australia	Turfsa	Algal turf mixed with sand (mostly turf)	https://coralnet.ucsd.edu/label/463/
Pacific Australia	Unc	Unclear	https://coralnet.ucsd.edu/label/118/
Southeast Asia	ALGAE_OTH	Other algae	https://coralnet.ucsd.edu/label/1607/
Southeast Asia	BRA_ARB_Ac	Branching *Acropora sp.*	https://coralnet.ucsd.edu/label/1408/
Southeast Asia	BRA_BLC	Bleached branching corals	https://coralnet.ucsd.edu/label/1416/
Southeast Asia	BRA_BOT_Ac	Branching *Acropora sp.:* Bottlebrush morphology (hispidose)	https://coralnet.ucsd.edu/label/1407/

Region	Label code	Label description and/or examples	URL
Southeast Asia	BRA_DIG_Ac	Digitate *Acropora* sp. *e.g. A. humulis*	https://coralnet.ucsd.edu/label/1411/
Southeast Asia	BRA_FIN_Se	Fine branching non-Acroporids: *Seriatopora* sp.	https://coralnet.ucsd.edu/label/1414/
Southeast Asia	BRA_OTH	Other branching corals: *Anacropora/Echinopora* (excluding *Pocillopora and Stylophora)*	https://coralnet.ucsd.edu/label/1415/
Southeast Asia	BRA_RND_St	Branching *Stylophora* sp.	https://coralnet.ucsd.edu/label/1410/
Southeast Asia	BRA_SMO_Po	Branching *Porites* sp.	https://coralnet.ucsd.edu/label/1413/
Southeast Asia	BRA_TAB-Ac	*Acropora* sp.: corymbose/tabular/plate (no digitate)	https://coralnet.ucsd.edu/label/1412/
Southeast Asia	BRA_VER_Po	*Pocillopora* sp.	https://coralnet.ucsd.edu/label/1409/
Southeast Asia	CAL_CCA_DC	Calcifying calcareous crustose algae: smothering reef surface	https://coralnet.ucsd.edu/label/1403/
Southeast Asia	CAL_CCA_RB	Calcifying calcareous crustose algae: smothering rubble	https://coralnet.ucsd.edu/label/1597/
Southeast Asia	CYANO_DHC	Cyanobacteria smothering reef surface	https://coralnet.ucsd.edu/label/1439/
Southeast Asia	CYANO_RB	Cyanobacteria smothering rubble	https://coralnet.ucsd.edu/label/1440/
Southeast Asia	EAM_DHC	Epilithic algal matrix: Multi-specific algal assemblage smothering reef surface 1 cm or less in height	https://coralnet.ucsd.edu/label/1432/
Southeast Asia	EAM_RB	Epilithic algal matrix: Multi-specific algal assemblage smothering rubble 1 cm or less in height	https://coralnet.ucsd.edu/label/1433/
Southeast Asia	EAM_Sub	Epilithic algal matrix on non-reef rock or other substrate	https://coralnet.ucsd.edu/label/1438/
Southeast Asia	FISH	Fish	https://coralnet.ucsd.edu/label/460/
Southeast Asia	GEAR	Fishing gear	https://coralnet.ucsd.edu/label/1431/
Southeast Asia	LSUB_RUB	Loose substrate: rubble	https://coralnet.ucsd.edu/label/1703/
Southeast Asia	LSUB_SAND	Loose substrate: sand	https://coralnet.ucsd.edu/label/1702/
Southeast Asia	LSUB_SEDI	Loose substrate: terrigenous sediments	https://coralnet.ucsd.edu/label/1704/
Southeast Asia	MACR_Cal_H	Calcifying macroalgae: *Halimeda* sp.	https://coralnet.ucsd.edu/label/1442/
Southeast Asia	MACR_Cal_P	Calcifying macroalgae: *Padina sp.*	https://coralnet.ucsd.edu/label/1443/
Southeast Asia	MACR_Fil_A	Filamentous macroalgae	https://coralnet.ucsd.edu/label/1448/
Southeast Asia	MACR_Fol_B	Foliose strap/branched macroalgae	https://coralnet.ucsd.edu/label/1650/
Southeast Asia	MACR_Fol_F	Foliose fan shaped macroalgae	https://coralnet.ucsd.edu/label/1446/
Southeast Asia	MACR_Fol_O	Other foliose macroalgae	https://coralnet.ucsd.edu/label/1605/
Southeast Asia	MACR_Fol_P	Foliose feathery macroalgae	https://coralnet.ucsd.edu/label/1651/
Southeast Asia	MACR_GLOB	Macroalgae: large visible globules	https://coralnet.ucsd.edu/label/1654/
Southeast Asia	MASE_BLC	Bleached Massive/Submassive/Encrusting corals	https://coralnet.ucsd.edu/label/1424/
Southeast Asia	MASE_LRG_I	*Isopora* sp.	https://coralnet.ucsd.edu/label/1595/
Southeast Asia	MASE_LRG_O	Massive Submassive Encrusting colonies with large visible rounded polyps: *Diploastrea; Favia; Favites; Montastraea; Galaxea; Goniopora; Astreopora*	https://coralnet.ucsd.edu/label/1593/
Southeast Asia	MASE_MEA_L	*Lobophyllia* sp.	https://coralnet.ucsd.edu/label/1418/
Southeast Asia	MASE_MEA_O	Massive Submassive Encrusting colonies with meandering ridges and valleys resembling brain: *Platygyra; Leptoria; Goniastrea*	https://coralnet.ucsd.edu/label/1417/
Southeast Asia	MASE_SML_O	Massive Submassive Encrusting colonies with small or invisible polyps (including columnar forms): *Pavona; Psammocora; Coscinaraea; Gardineroseris*	https://coralnet.ucsd.edu/label/1594/
Southeast Asia	MASE_SMO_P	Family Poritidae; massive/sub-massive morphology e.g. *P. lobata; P. lutea*	https://coralnet.ucsd.edu/label/1596/
Southeast Asia	MINV_COTS	Crown-of-thorns starfish	https://coralnet.ucsd.edu/label/1648/
Southeast Asia	MINV_CRI	Crinoids	https://coralnet.ucsd.edu/label/1649/
Southeast Asia	MINV_OTH	Other mobile invertebrates: sea cucumbers/sea urchins/sea stars/lobster	https://coralnet.ucsd.edu/label/1455/
Southeast Asia	NON_FREE	Non hermatypic corals: Free living (*Fungia* etc.)	https://coralnet.ucsd.edu/label/1429/
Southeast Asia	NON_HEL	Non hermatypic: *Heliopora*	https://coralnet.ucsd.edu/label/1754/
Southeast Asia	NON_MIL	Non Hermatypic: *Millepora*	https://coralnet.ucsd.edu/label/1655/
Southeast Asia	SINV_HEX_O	Other sesile invertebrates soft hexacorallia	https://coralnet.ucsd.edu/label/1454/
Southeast Asia	SINV_HYD	Hydroids feathery types	https://coralnet.ucsd.edu/label/1602/
Southeast Asia	SINV_OTH	Other sesile invertebrates: Bryozoa, clams	https://coralnet.ucsd.edu/label/1437/

Region	Label code	Label description and/or examples	URL
Southeast Asia	SINV_SFC_A	Soft coral; Common Alcyoniidae: *Lobophytum; Sarcophyton*	https://coralnet.ucsd.edu/label/1434/
Southeast Asia	SINV_SFC_E	Sea fans/plumes/branching whipes	https://coralnet.ucsd.edu/label/1436/
Southeast Asia	SINV_SFC_O	Other soft-corals no common Alcyoniidae	https://coralnet.ucsd.edu/label/1459/
Southeast Asia	SINV_SPO_E	Sponges: Branching/rope forms	https://coralnet.ucsd.edu/label/1601/
Southeast Asia	SINV_SPO_F	Sponges: Fan shaped forms	https://coralnet.ucsd.edu/label/2092/
Southeast Asia	SINV_SPO_M	Sponges: Massive or encrusting	https://coralnet.ucsd.edu/label/1405/
Southeast Asia	SINV_SPO_V	Sponges: Hollow sponge forms/cups/barrels/tub	https://coralnet.ucsd.edu/label/1456/
Southeast Asia	SINV_TUN	Tunicates (ascidians)	https://coralnet.ucsd.edu/label/1603/
Southeast Asia	TFP_RDG_Al	Thin, Foliose, and Plate colonies (excluding *Acropora, Porites*) with/without visible relief structure: e.g. *Echinophyllia; Turbinaria; Montipora; Echinopora*	https://coralnet.ucsd.edu/label/1426/
Southeast Asia	TFP_RND_Al	Thin, Foliose, and Plating (except Acroporids) colonies with visible rounded corallites on the plates: Includes *Turbinaria, Echinopora*	https://coralnet.ucsd.edu/label/1427/
Southeast Asia	TFP_SMO_Po	Family Poritidae; encrusting morphology e.g. *P. lichen*	https://coralnet.ucsd.edu/label/1425/
Southeast Asia	TSH	Trash: Human Origin	https://coralnet.ucsd.edu/label/1600/
Southeast Asia	Unc	Unclear	https://coralnet.ucsd.edu/label/118/
Southeast Asia	Unk	Unknown	https://coralnet.ucsd.edu/label/1647/

References

1 González-Rivero, M. *et al.* Monitoring of Coral Reefs Using Artificial Intelligence: A Feasible and Cost-Effective Approach. *Remote Sensing* **12**, 489, doi:https://doi.org/10.3390/rs12030489 (2020).

2 González-Rivero, M. *et al.* Scaling up Ecological Measurements of Coral Reefs Using Semi-Automated Field Image Collection and Analysis. *Remote Sensing* **8**, 30, doi:https://doi.org/10.3390/rs8010030 (2016).

3 González-Rivero, M. *et al.* The Catlin Seaview Survey - kilometre-scale seascape assessment, and monitoring of coral reef ecosystems. *Aquatic Conservation: Marine and Freshwater Ecosystems* **24**, 184-198, doi: https://doi.org/10.1002/aqc.2505 (2014).
